# In silico analysis of the wheat *BBX* gene family and identification of candidate genes for seed dormancy and germination

**DOI:** 10.1186/s12870-024-04977-x

**Published:** 2024-04-25

**Authors:** Xinran Cheng, Shuying Lei, Jin Li, Bingbing Tian, Chunxiu Li, Jiajia Cao, Jie Lu, Chuanxi Ma, Cheng Chang, Haiping Zhang

**Affiliations:** 1 College of Agronomy, Key Laboratory of Wheat Biology and Genetic Improvement on Southern Yellow & Huai River Valley, Ministry of Agriculture and Rural Affairs, Anhui Agricultural University, Hefei, Anhui 230036 China; 2 National Key Laboratory for Crop Genetics and Germplasm Enhancement, Jiangsu Plant Gene Engineering Research Center, Nanjing Agricultural University, Nanjing, 210095 China

**Keywords:** Wheat, B-box protein, Phylogenetic relationship, Seed dormancy and germination, Pre-harvest sprouting

## Abstract

**Background:**

B-box (BBX) proteins are a type of zinc finger proteins containing one or two B-box domains. They play important roles in development and diverse stress responses of plants, yet their roles in wheat remain unclear.

**Results:**

In this study, 96 *BBX* genes were identified in the wheat genome and classified into five subfamilies. Subcellular localization prediction results showed that 68 *TaBBXs* were localized in the nucleus. Protein interaction prediction analysis indicated that interaction was one way that these proteins exerted their functions. Promoter analysis indicated that *TaBBXs* may play important roles in light signal, hormone, and stress responses. qRT-PCR analysis revealed that 14 *TaBBXs* were highly expressed in seeds compared with other tissues. These were probably involved in seed dormancy and germination, and their expression patterns were investigated during dormancy acquisition and release in the seeds of wheat varieties Jing 411 and Hongmangchun 21, showing significant differences in seed dormancy and germination phenotypes. Subcellular localization analysis confirmed that the three candidates TaBBX2-2 A, TaBBX4-2 A, and TaBBX11-2D were nuclear proteins. Transcriptional self-activation experiments further demonstrated that TaBBX4-2A was transcriptionally active, but TaBBX2-2A and TaBBX11-2D were not. Protein interaction analysis revealed that TaBBX2-2A, TaBBX4-2A, and TaBBX11-2D had no interaction with each other, while TaBBX2-2A and TaBBX11-2D interacted with each other, indicating that TaBBX4-2A may regulate seed dormancy and germination by transcriptional regulation, and TaBBX2-2A and TaBBX11-2D may regulate seed dormancy and germination by forming a homologous complex.

**Conclusions:**

In this study, the wheat BBX gene family was identified and characterized at the genomic level by bioinformatics analysis. These observations provide a theoretical basis for future studies on the functions of *BBXs* in wheat and other species.

**Supplementary Information:**

The online version contains supplementary material available at 10.1186/s12870-024-04977-x.

## Introduction

Transcription factors (TFs) are important gene regulators widely involved in the regulation of growth, development, metabolism, and environmental responses. Zinc finger proteins (ZFPs) are a group of TFs with finger-like domains (composed of histidine, cysteine, and zinc ions). Zinc finger domains can interact with DNA, RNA and proteins, regulating transcription, RNA packaging, cell apoptosis, and protein folding and assembly. ZFPs can be divided into several subfamilies due to their structural diversity [1].

B-box (BBX) is a subfamily of ZFPs that contain one or two B-box domains in their amino acid sequence, and some BBXs also contain the conserved carboxy terminal (CCT), CONSTANS (CO), CO-like, translocon the outer membrane of the chloroplast (TOC), VP (G-I/V-V-P-S/T-F), and other domains at the C-terminus [2]. B-box conserved domains are important in protein interactions and transcriptional regulation, CCT domains are mainly involved in nuclear transport and transcriptional regulation, and VP motifs (generally 16–20 amino acid residues from the CCT domain) are important in BBX/coil protein interaction [2]. Plant BBXs are further classified into five categories based on their number of B-box domains and the presence or absence of the CCT domain [2]. Types I and II contain two B-box domains and one CCT domain (B1 + B2 + CCT), type III contains one B-box domain and one CCT domain (B1 + CCT), type IV contains two B-box domains (B1 + B2), and type V contains one B-box domain (B1). The amino acid sequence of B2 is different in types I and II.

BBX proteins have undergone substantial research in eukaryotes. In mammals, the B-box domain frequently produces trivalent structural proteins (e.g., tripartite motif/ RING-B-box-coiled-coil), which perform roles in the cellular ubiquitination cascade, protein transport, and transcriptional control [2]. In comparison to animal research, functional investigations on BBX proteins in plants are rather late.

The plant *B-box* gene *CO*, was first identified in 1995 in a late-flowering *Arabidopsis* mutant [3]. Subsequent studies showed that plant *BBXs* were involved in important growth and development processes, including photomorphogenesis, flowering physiology, shade effects, hormone signaling, and stress responses. *BBXs* promoting photomorphogenesis often act downstream of various phytochrome and cytochrome pathways [2]. For example, *AtBBX21* bound to the ELONGATED *HYPOCOTYL 5* (*HY5*) promoter, enhanced its expression activity, and participated in seedling photomorphogenesis [4]. *AtBBX25* negatively regulated plant photomorphogenesis by dimerization with *HY5* and inhibiting its transcriptional activity [2]. *SlBBX20* in *Solanum lycopersicum* bound to the promoter of *PHYTOENE SYNTHASE 1*, a key carotenoid synthesis enzyme gene, induced its expression, and participated in carotenoid synthesis, thereby participating in photomorphogenesis [5]. MdBBX37 in apple interacted with MdMYB1 and MdMYB9, inhibiting the binding of these two proteins to their target genes, and thus negatively regulating anthocyanin biosynthesis and hypocotyl elongation [6]. Apple MdPIF7 regulated anthocyanin biosynthesis and hypocotyl growth by interacting with MdBBX23 and attenuating the transcriptional activation of *MdBBX23* on *MdHY5* [7].

Several conditions control plant flowering, among which the effect of photoperiod on plant flowering is intimately associated with *BBXs* [2, 8, 9]. For example, in *Arabidopsis*, CO/AtBBX1 directly bound to the *FLOWERING LOCUS T* (*FT*) promoter and activated *FT* expression to promote plant flowering under long-day conditions [10, 11]. AtBBX32 interacted with EMBRYONIC FLOWER1 (EMF1) to regulate flowering time. AtBBX6 promoted the expression of *FT* and *SOC* under short-day conditions, and the overexpression of *AtBBX6* in the *co*-mutant can also suppress the late flowering effect to some extent [12]. Flowering was also affected by *AtBBX19* and *AtBBX13* (*COL12*). AtBBX19 can compete with CO to bind to the *FT* promoter, suppressing the expression of *FT* and its downstream genes, thereby inhibiting flowering. On the other hand, COL12 affected flower development by interacting with CO and altering CO activity. In rice, the *CO* homologous gene *Heading date 1* (*Hd1*) formed a protein complex with haloolerance protein 3 (OsHAL3) or grain number, plant height and heading date 7 (GHD7) to promote short-day flowering and inhibit long-day flowering [13]. OsBBX27 affected flowering by down-regulating *Hd3a* and *FLOWERING LOCUS T-like* (*FTL*) expression [14]. OsCOL15 affected flowering by up-regulating *GHD7* expression or down-regulating *rice indeterminate 1* (*RID1*) expression [15]. In barley, HvCO1 promoted florigenesis by up-regulating the *HvFT* expression. In *Brassica rapa*, BrBBX32 promoted flowering by interacting with AGAMOUS-LIKE 24 (BrAGL24) via its B-box domain [8].

*BBXs* play certain roles in abiotic stress response [2]. For example, *AtBBX31* was involved in promoting photomorphogenesis in response to UV-B radiation and increased tolerance to high doses of UV-B radiation [16]. *AtBBX24* (originally known as *STO)* was involved in salt stress signaling and conferred increased salt tolerance in salt-sensitive mutant yeast [17]. *AtBBX5* was involved in stress response through the abscisic acid (ABA) signaling pathway, and its expression was up-regulated in response to exogenous ABA, salt, and osmotic stress [18]. *AtBBX18* down-regulated expression increased heat tolerance, and the overexpression of this gene decreased the heat tolerance of plants [19]. *MdBBX10* increased salt and drought tolerance in *Arabidopsis*, and *MdBBX* increased cellular tolerance to salt and osmotic stress in *Escherichia. coli* [20].

*BBXs* have been identified in *Arabidopsis*, rice, tomato, pear, potato, grape, pear, peanut, etc [2, 21–23]. . , but not in wheat. Wheat (*Triticum aestivum* L.) is one of the most important food crops, representing about 30% of the cereal cultivated land in the world [24]. The release of the wheat genome made it possible to analyze the *TaBBX* gene family. Its genome is complex, formed by the integration of three sub-genomes A, B, and D into a heterohexaploid (AABBDD) containing up to 85% repetitive sequences and measuring approximately 17 Gb [25]. Low-dormancy wheat varieties are susceptible to pre-harvest sprouting (PHS) during the harvest season when rainfall is heavy and humidity is high [25]. Wheat PHS is a complex process involving many influencing factors, including external factors (temperature, humidity, light, etc.) and internal factors (seed moisture content, α-amylase activity, endogenous hormones, etc.), while seed dormancy is the main genetic mechanism regulating PHS resistance [26]. Wheat PHS is a difficult problem to solve worldwide. It is feasible to grow wheat varieties with strong dormancy to cope with long-period rainfall by the gene pyramiding approach, but the breeding cycle is too long. Therefore, the development of strong dormancy wheat varieties can be accelerated by exploring candidate genes and elucidating their regulatory mechanisms. Both AtBBX19 and AtBBX21 were reported to affect seed germination [4, 27]. AtBBX19 induced *ABI5* and inhibited seed germination. AtBBX21 directly activated *HY5* and was targeted by *COP1* for *26 S* proteasome-mediated degradation to promote seed germination. These results demonstrate that *Arabidopsis BBXs* play an important role in regulating seed germination. However, the role of *TaBBXs* in wheat seed dormancy and germination has not yet been reported.

In this study, the wheat *BBX* gene family was identified and characterized at the genomic level by bioinformatics analysis. The phylogenetic relationships between members of *BBX* gene family of rice, *Arabidopsis* and wheat were elucidated using the comparative genomics approach. The candidate *TaBBXs* associated with seed dormancy and germination were screened by expression analysis. Subcellular localization, transcriptional activity and interaction analysis of three representative candidate genes (*TaBBX2-2A/-4–2A/-11-2D*) were also performed. These results may serve as a theoretical basis for in-depth analysis of *BBX* functions, as well as for the genetic improvement of wheat PHS resistance.

## Results

### Identification of *TaBBX* genes

A total of 96 *TaBBXs*, named *TaBBX1-2A-TaBBX96-Un* according to their relative positions on chromosomes, were obtained with reference to the reported Pfam number (PF00643) of *AtBBXs* and *OsBBX*s (Table S1). *TaBBXs* varied widely in length from 441 (*TaBBX27-5A*) to 1473 (*TaBBX73-7A*) bp and molecular weight (MW) from 15371.44 (*TaBBX27-5A*) to 52005.04 (*TaBBX73-7A*) Da. The isoelectric points (pI) of 96 *TaBBXs* ranged from 4.32 to 10.42, with 82 *TaBBXs* pI less than 7 (acidic amino acids) and 14 *TaBBXs* pI greater than 7 (basic amino acids). None of the *TaBBXs* encoded pyrrolysine (Pyl) and selenium cysteine (Sec) (Table S2). The instability indexs (II) ranged from 37.87 to 70.35, with only TaBBX68-7A protein being less than 40. The grand average of hydropathicity (GRAVY) values ranged from − 0.713 to 0.286, and only nine TaBBXs proteins had GRAVY greater than 0. The aliphatic indexs (AI) were between 54.18 and 100.91 (Table S1).

### Phylogenetic analysis of the *BBX* gene family

**Fig. 1:**
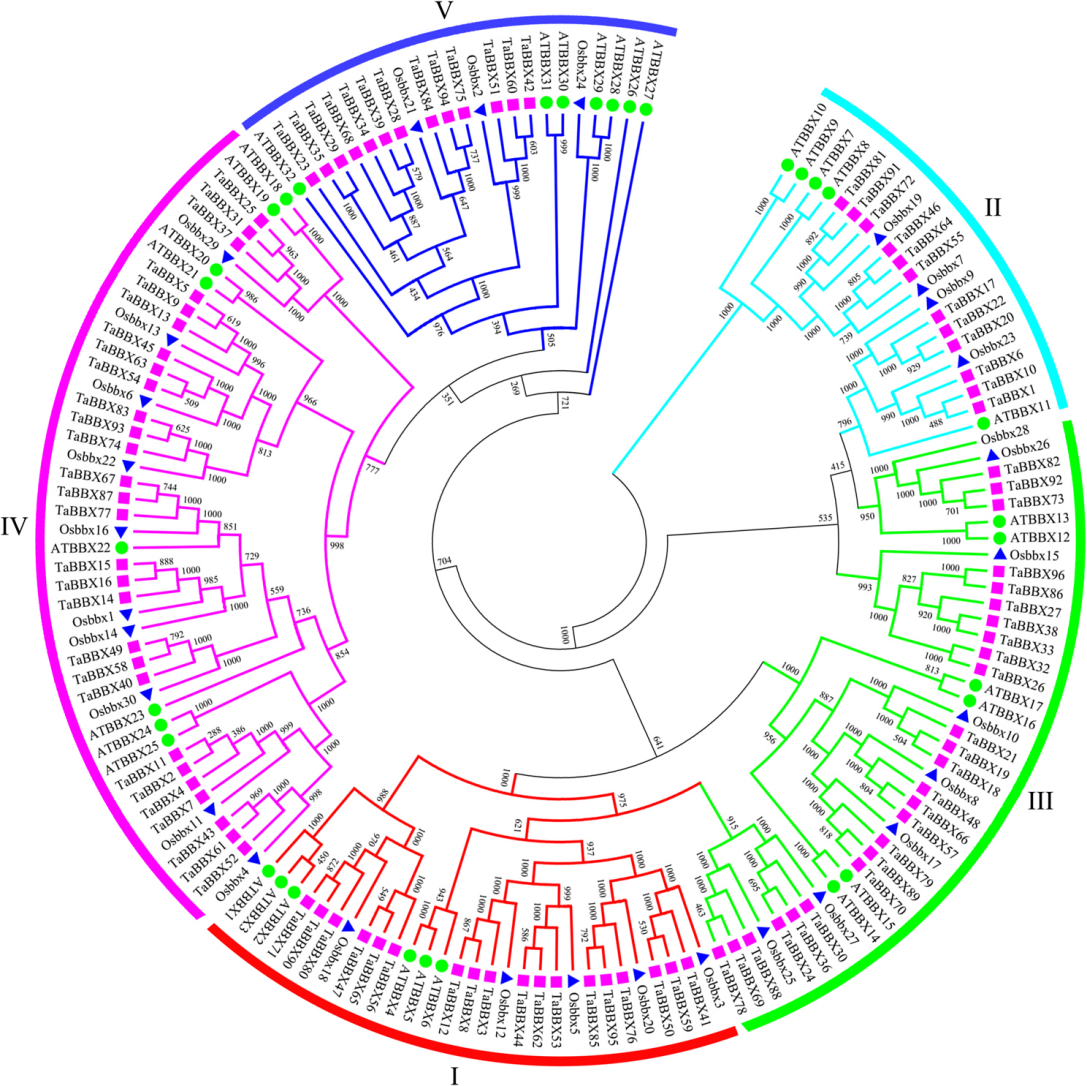
Phylogeny of *BBXs* from wheat, rice, and *Arabidopsis.* The 96 *TaBBXs*, 30 *OsBBXs*, and 32 *AtBBXs*, were clustered into five subfamilies. Details of *BBXs* from *Arabidopsis* and rice were listed in Table S3

A phylogenetic tree was constructed for 96 *TaBBXs*, 32 *AtBBXs* and 30 *OsBBXs*, to investigate the phylogenetic relationships and functional differences in *TaBBXs* members in detail (Table S3). *TaBBXs* were further divided into five subfamilies (I-V) with 18, 12, 25, 28 and 13 members, respectively, based on the grouping and analysis of Khanna et al. (2009) (Fig. 1) [25]. Subfamily I contained two B-box domains and one CCT domain, while subfamily IV lacked a CCT domain but contained two B-box domains. In addition, some TaBBXs contained other domains, such as VP, CO, CO-like and TOC.

Thirty-six homologous pairs were identified, including two paralogous *Ta-Ta* pairs, one orthologous *Ta-At* pair and 33 orthologous *Ta-Os* pairs (Table S4). Furthermore, Ka, Ks and Ka/Ks values were calculated for the 36 homologous pairs (Table S4), and 13 homologous pairs had Ka/Ks ratios < 1 and 23 pairs had Ka/Ks ratios > 1 (Fig.S1). We also analyzed the homologous *TaBBX* genome (Table S5), and 77 genes (90.63%) were present as triplets, and a homologous gene was frequently lost (6.25%), whereas only a single gene was rarely lost (3.12%).

### Gene structure analysis of the wheat *BBX* family

Ninety-five *TaBBXs* were unevenly distributed on 18 of 21 wheat chromosomes, with none found on chromosomes 1A, 1B or 1D. *TaBBX96-Un* was mapped to a non-distributed scaffold and could not be further located on the chromosome (Fig.S2). The largest gene numbers were distributed on chr7 containing 29 genes, followed by chr6 containing 27 genes. The lowest gene numbers were distributed on chr3 containing three genes. There were 51 *TaBBXs* that formed 22 gene pairs clusters, with chr6 containing nine clusters (21 *TaBBXs*), chr7 containing seven clusters (14 *TaBBXs*), and chr2 (6 *TaBBXs*) and chr5 (10 *TaBBXs*) containing three clusters each (Fig.S2).

*TaBBXs* exon numbers ranged from one to six, and intron numbers ranged from zero to four (Table S1, Fig.S3). Most *TaBBXs* contained two exons (37.5%), and only one gene had six exons (*TaBBX55-6B*, subfamily II). Gene structure analysis showed that subfamily V members had no introns, most subfamily I members had one intron (except for *TaBBX41-6A/-50-6B/-59-6D*), and *TaBBX20-4B/-22-4D/-46–6A* in subfamily II contained two introns, *TaBBX20-4B* contained four introns, while the remaining of the genes contained three introns. We found that two paralogous pairs had the same number of exons. We also found that 24 triplets (82.76%) had the same number of exons.

TaBBX proteins length ranged from 146 (TaBBX27-5A) to 490 (TaBBX73-7A) amino acids (Table S1). Multiple sequence alignment showed that 25 TaBBXs had two B-box domains and one CCT domain, 29 TaBBXs had two B-box domains but no CCT domain, 15 TaBBXs contained only one B-box domain, and 27 TaBBXs contained one B-box domain and one CCT domain (Fig.S4, Table S6).

Subcellular localization prediction revealed that TaBBXs were mainly localized in the nucleus (68, 70.83%), followed by the extracellular (12, 12.5%), and least localized in the cytoplasm (6, 62.5%) (Table S7, Fig.S5A). Of these, subfamily II members were localized only in the nucleus, and subfamily V members were localized only in the nucleus and extracellular matrix. Furthermore, signal peptide analysis results showed that signal peptides were present in only 13 genes, mostly in subfamily III (9, 69.23%) (Table S8, Fig.S5B). No signal peptide was present in subfamily II members, and the rest were evenly distributed in subfamilies I, IV, and V.

MiRNA analysis indicated that 18 *TaBBXs* were targeted by seven miRNAs (*tae-miR171b*, *tae-miR397-5p*, *tae-miR408*, *tae-miR9657a-3p*, *tae-miR9661-5p*, *tae-miR9780*, and *tae-miR399*) (Table S9, Fig.S5C). *tae-miR9780* could target six genes, and *TaBBX67-7A* was targeted by both *tae-miR9657a-3p* and *tae-miR9661-5p*.

In protein interaction prediction, 36 genes were detected, of which *TaBBX14-3A/-44–6A/-49-6B/-51-6B/-86-7D/-96-Un* had no interactions, and all other genes had interactions with multiple genes (Fig.S6). The types of interactions can be divided into two categories, the first is Known Interactions, where one type has been experimentally determined, e.g. *TaBBX9-2B-TaBBX23-4D*, and the second is Others, which can be divided into three types, i: textmining, e.g. *TaBBX15-3B-TaBBX74-7A*; ii: co-expression, e.g. *TaBBX55-6B-TaBBX81-7B*; and iii: protein homology, e.g. *TaBBX2-2A-TaBBX25-5A*. Some interacting gene pairs existed for all four relationships, e.g. *TaBBX25-5A-TaBBX47-6A*.

### Promoter and GO analysis of the *BBX* gene family

**Fig. 2:**
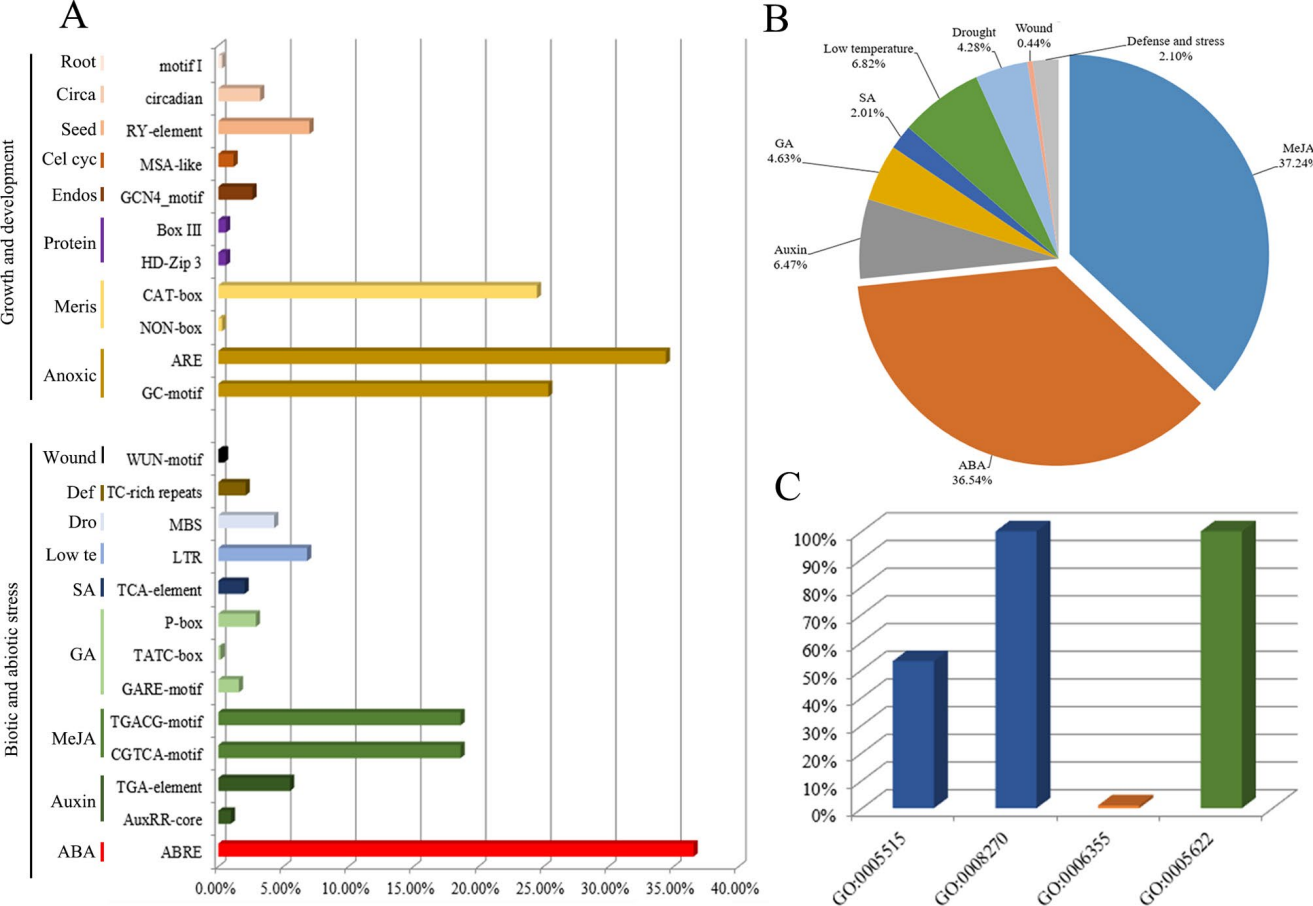
Promoter and GO analysis of *TaBBXs*. **A**. Percentage of cis-acting element in the promoter regions of *TaBBXs.* Abbreviations represent: Root, Root specific; Circa, Circadian control; Seed, Seed regulation; Cell cyc, Cell cycle regulation; Endos, Endosperm expression; Protein, Protein binding; Meris, Meristem expression; Anoxic, Anoxic inducibility; Def, defense and stress; Dro, Drought; Low te, Low temperature; SA, salicylic acid; GA, gibberellin; MeJA, Methyl jasmonate; ABA, abscisic acid. **B**. Percentage of biotic and abiotic stress response elements in the promoter region of *TaBBXs*. **C**. Gene Ontology (GO) results in *TaBBXs*. Different colors represent different functions

Forty-eight *cis*-acting elements were identified in *TaBBXs*, and these can be divided into three groups (Table S10). The first group was light response elements, including 22 types of G-box, ACE, AE-box, Sp1, GT1-motif, GATA-motif, GA-motif, etc. The second group was biotic and abiotic stress response elements, including 13 types of CGTCA-motif, TGACG-motif, ABRE, MBS, LTR, etc. The third group was growth and development response elements, including 13 types such as GCN4_motif, ARE, CAT-box, etc. (Fig. 2A). A total of 1144 *cis*-acting elements were identified in second group, with the largest number of methyl jasmonic acid (MeJA) response elements (CGTCA-motif and TGACG-motif) (426, 37.24%), followed by ABA response elements (ABRE, 418, 36.54%). A total of 343 *cis*-acting elements were identified in third group, with the largest number of anaerobic induction response elements (ARE and GC-motif) (205, 59.76%), followed by meristem expression response elements (CAT-box, 84, 25.36%) and seed-specific regulation response elements (RY-element, 24, 7%) (Fig. 2B).

In GO analysis, 96 *TaBBXs* contained four GO annotations, which could be divided into three categories (Table S11, Fig. 2C) of molecular_function (GO:0005515 and GO:0005622), cellular_component (GO:0008270) and biological_process (GO:0006355). All *TaBBXs* contained GO:0005622 (intracellular anatomical structure) and GO:0008270 (zinc ion binding) annotations, and only *TaBBX86-7D* had all four annotations present.

### Expression profiling and co-regulatory networks analysis of the *BBX* gene family

In GSE12508 data, expression data of 57 *TaBBXs* were obtained from 13 tissues (GSC, germinating seed coleoptile; GSR, germinating seed root; GSE, germinating seed embryo; SR, seedling root; SC, seedling crown; SL, seedling leaf; II, immature inflorescence; Fba, floral bracts before anthesis; Pba, pistil before anthesis; Aba, Anthers before anthesis; 3–5 DAP C, 3–5 DAP caryopsis; 22 DAP EN, 22 DAP endosperm; 22 DAP EM, 22 DAP embryo) (Table S12, Fig.S7A). Twenty *TaBBXs* (*TaBBX24-5A/-40–6A/-42–6A/-47–6A/-49-6B/-51-6B/-56-6B/-58-6D/-60-6D/-64-6D/-65-6D/-70–7A/-71–7A/-74–7A/-79-7B/-80-7B/-83-7B/-89-7D/-90-7D/-93-7D*) were lowly expressed in all tissues, six *TaBBXs* (*TaBBX25-5A/-31-5B/-37-5D/-67–7A/-77-7B/-87-7D*) were highly expressed in all tissues, nine *TaBBXs* (*TaBBX3-2A/-12-2D/-14–3A/-15-3B/-16-3D/-18–4A/-19-4B/-21-4D/-66-6D*) were highly expressed in Fba and SL, four *TaBBXs* (*TaBBX2-2A/-4–2A/-7-2B/-11-2D*) were highly expressed in Fba, SL, 3–5 DAP C, Pba and Aba, and three *TaBBXs* (*TaBBX43-6A/-52-6B/-61-6D*) were lowly expressed in 22 DAP-EN, 3–5 DAP C, Pba, SR and SC.

In GSE49821 data, expression data of 57 *TaBBXs* were obtained from JM20 at different imbibition times (0, 12, 36, and 48 h) (Table S12, Fig.S8A). Twenty-five *TaBBXs* were expressed at low levels during seed imbibition, and nine *TaBBXs* (*TaBBX41-6A/-43–6A/-50-6B/-52-6B/-59-6D/-61-6D/-67–7A/-77-7B/-87-7D*) were highly expressed during seed imbibition. Of these, 19 *TaBBXs* (*TaBBX2-2A/-4–2A/-7-2B/-9-2B/-11-2D/-17–4A/-20-4B/-22-4D/-40–6A/-47–6A/-49-6B/-56-6B/-58-6D/-65-6D/-71–7A/-72–7A/-80-7B/-81-7B/-90-7D*) showed reduced expression with increasing imbibition time.

In GSE118474 data, expression data of 43 *TaBBXs* were obtained from four tissues (G-EB, 14 d Embryo; M-EB, 25 d Embryo; G-ES, 14 d Endosperm; M-ES, 25 d Endosperm) of Zhou8425b2 (Table S12, Fig.S9A). Eleven *TaBBXs* (*TaBBX7-2B/-9-2B/-23-4D/-26–5A/-29–5A/-39-5D/-45–6A/-47–6A/-68–7A/-70–7A/-78-7B*) were expressed at low levels in all tissues, and five *TaBBXs* (*TaBBX67-7A/-72–7A/-77-7B/-81-7B/-87-7D*) were highly expressed in all tissues. In addition, nine *TaBBXs* (*TaBBX1-2A/-3–2A/-8-2B/-17–4A/-21-4D/-30-5B/-36-5D/-55-6B/-64-6D*) were expressed more in M EB and M EN than in G EB and G EN, and 17 *TaBBXs* (*TaBBX5-2A/-14–3A/-15-3B/-31-5B/-37-5D/-40–6A/-41–6A/-43–6A/-44–6A/-59-6D/-63-6D/-71–7A/-73–7A/-76–7A/-82-7B/-84-7B/-85-7B*) were less expressed in M EB and M EN than in G EB and G EN.

We performed a co-regulatory network analysis on the genes in three types of GEO data and found that *TaBBXs* had varying degrees of positive correlation (Fig.S7B-C, Fig.S8B-C and Fig.S9B-C).

### Expression profiles and co-regulatory networks of subfamily IV *TaBBXs* in different tissues and imbibition time points

**Fig. 3:**
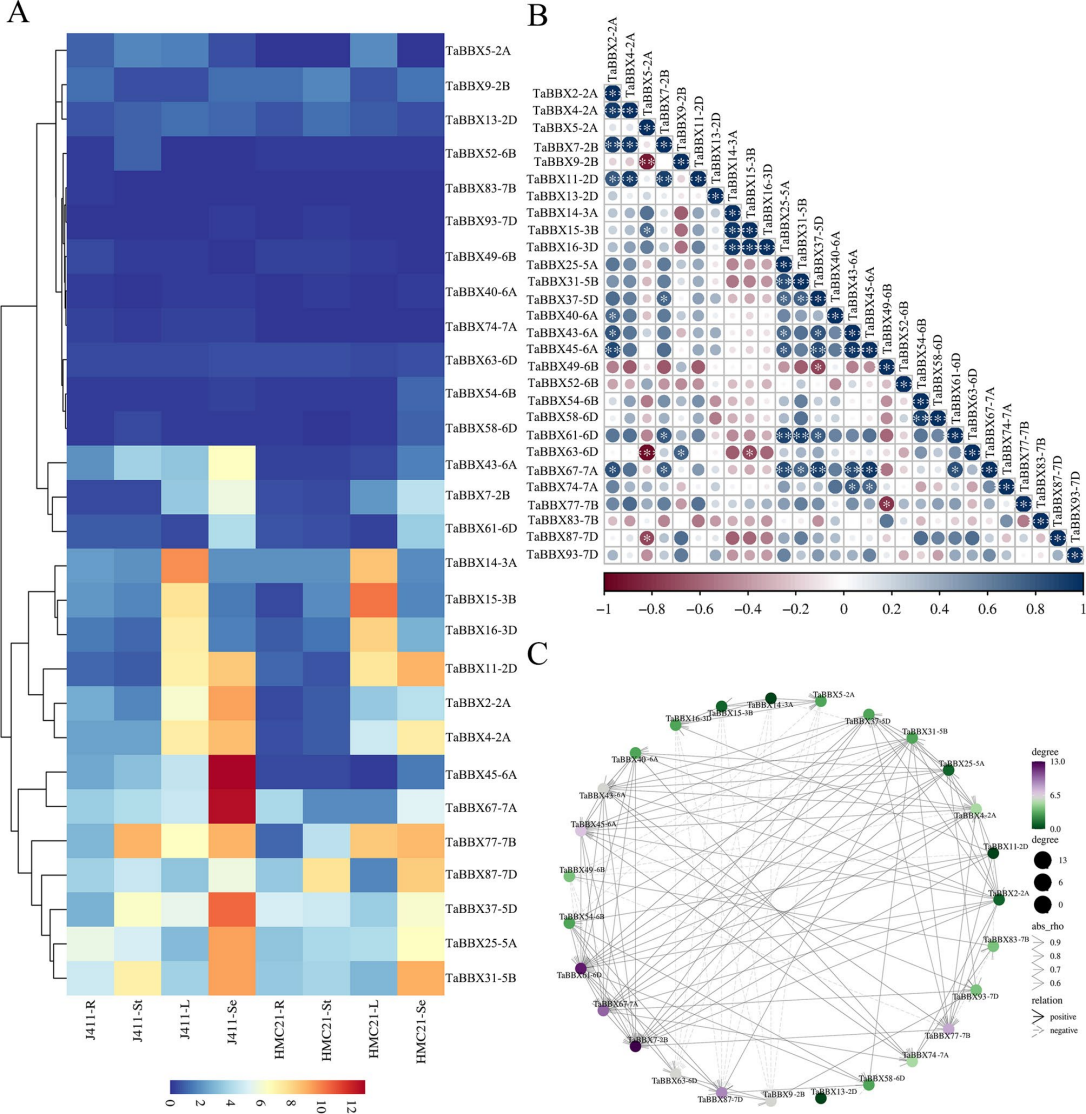
Expression patterns of *TaBBXs* in different wheat tissues by qRT-PCR. **A**. Heatmap shows the hierarchical clustering of *TaBBXs* in different tissues of wheat varieties Jing 411 (J411) and Hongmangchun 21 (HMC21). Three biological replicates per organization. Abbreviations represent tissue samples collected from J411 and HMC21: J411-R, J411-Root; J411-St, J411-Stem; J411-L, J411-Leaf; J411-Se, J411-Seed; HMC21-R, HMC21-Root; HMC21-St, HMC21-Stem; HMC21-L, HMC21-Leaf; HMC21-Se, HMC21-Seed. **B**. Correlation analysis using the R package program. Each correlation is shown by the shades of blue and red and the size of the circle shape. Blue and red indicate a positive correlation and negative correlation, respectively. **C**. Co-regulatory networks. The co-regulatory networks of *TaBBXs* were established based on the PCCs of these gene pairs using transformed qPCR data. Different colors and styles indicate the different significance levels of the co-regulated gene pairs

**Fig. 4:**
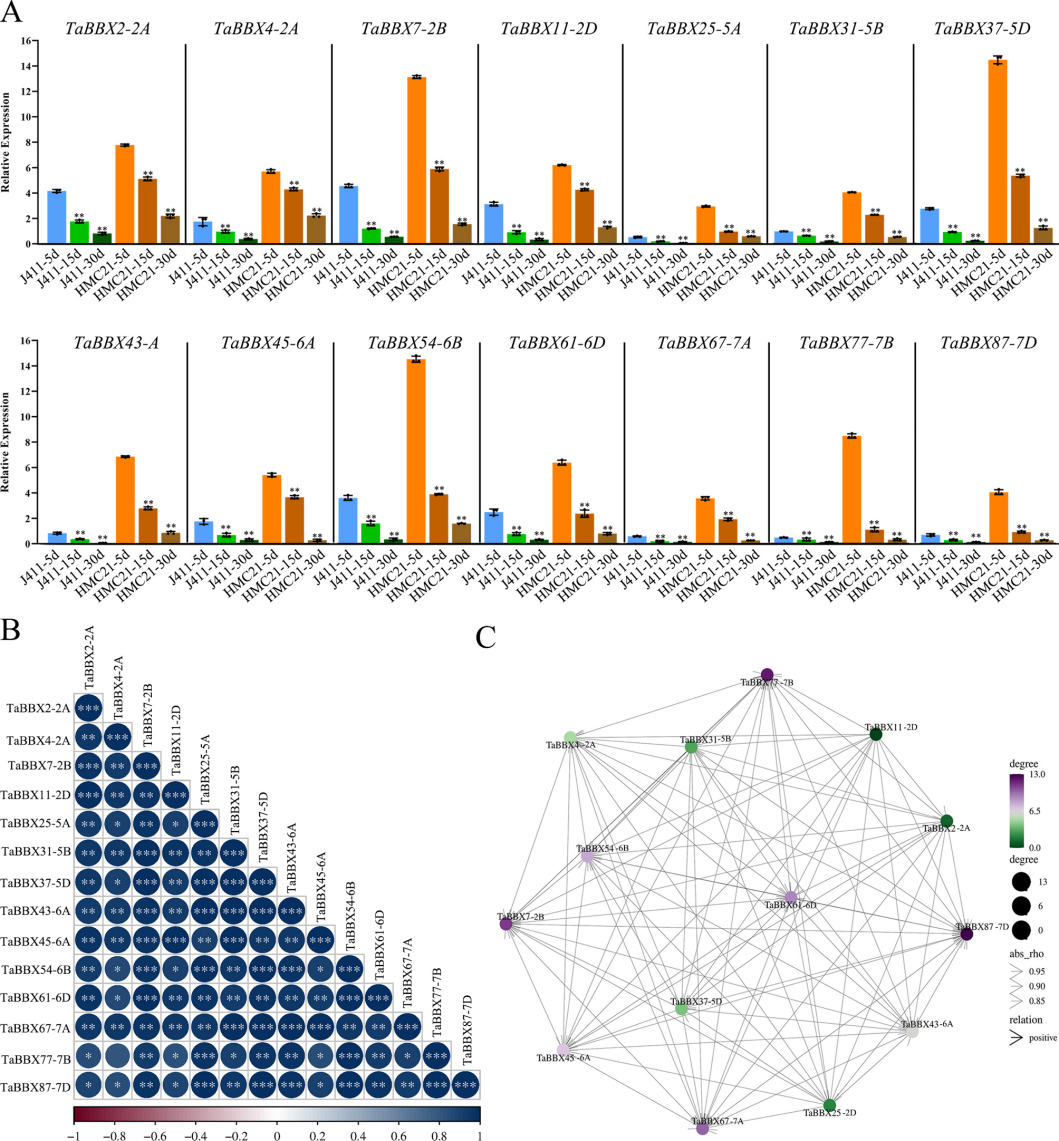
Expression patterns of *TaBBXs* at different seed development stages by qRT-PCR. **A**. Expression patterns of *TaBBXs* at the 3w/4w/5w post anthesis. Three biological replicates per organization. Abbreviations represent: J411-3 W, J411-3 week; J411-4 W, J411-4 week; J411-5 W, J411-5 week; HMC21-3 W, HMC21-3 week; HMC21-4 W, HMC21-4 week; HMC21-5 W, HMC21-5 week. **B**. Correlation analysis using the R package program. Each correlation is shown by the shades of blue and red and the size of the circle shape. Blue and red indicate a positive correlation and negative correlation, respectively. **C**. Co-regulatory networks. The co-regulatory networks of *TaBBXs* were established based on the PCCs of these gene pairs using transformed qPCR data. Different colors and styles indicate the different significance levels of the co-regulated gene pairs

**Fig. 5:**
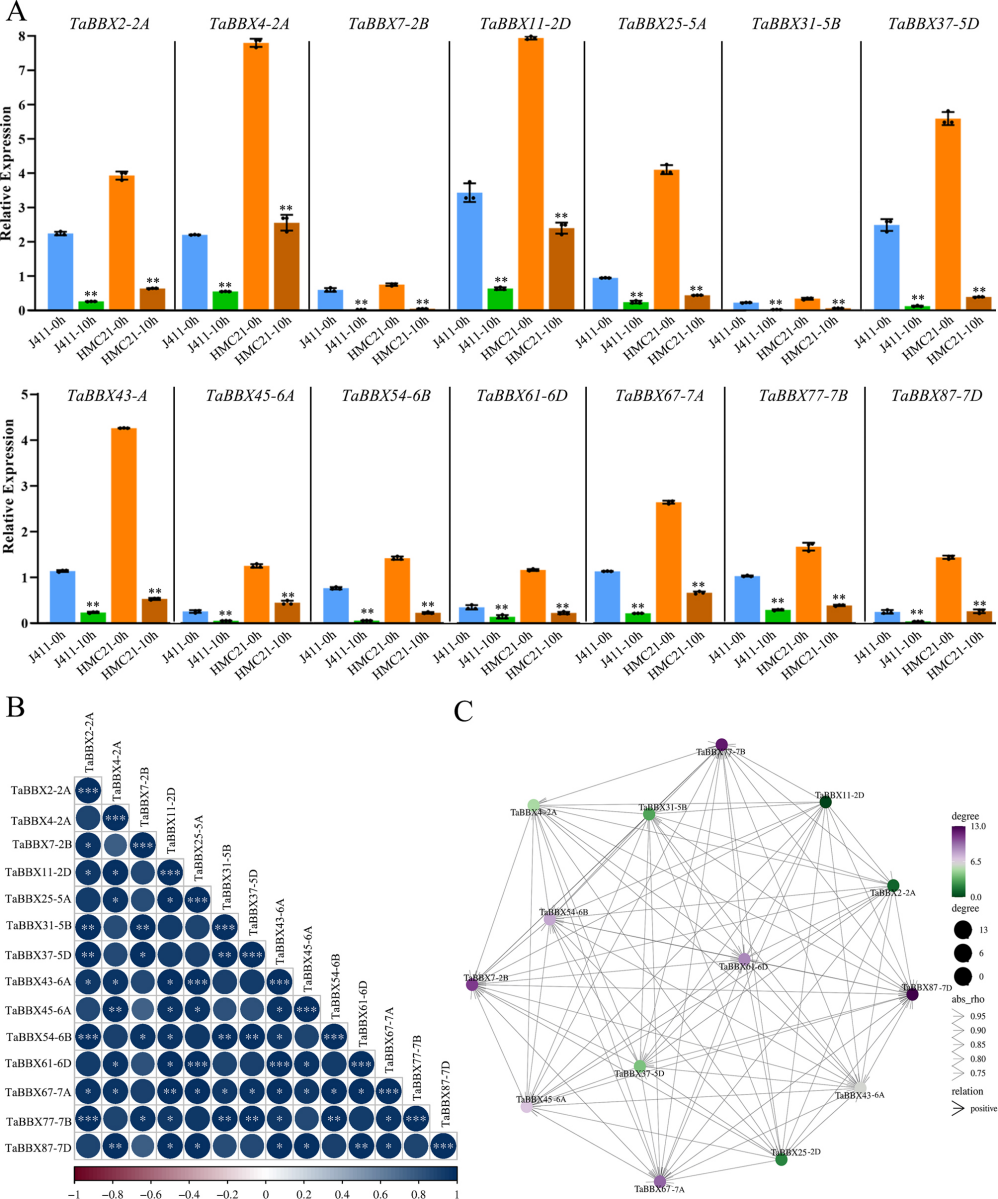
Expression patterns of *TaBBXs* at different post-harvest stages by qRT-PCR. **A**. Expression patterns of *TaBBXs* at 5, 15, and 30d post-harvest in the seeds of two wheat varieties Jing 411 (J411) and Hongmangchun 21 (HMC21). Three biological replicates per organization. Abbreviations represent the seed samples of J411 and HMC21 collected at different developmental stages: J411-5d, J411-5 day; J411-15d, J411-15 day; J411-30d, J411-30 day; HMC21-5d, HMC21-5 day; HMC21-15d, HMC21-15 day; HMC21-30d, HMC21-30 day. **B**. Correlation analysis using the R package program. Each correlation is shown by the shades of blue and red and the size of the circle shape. Blue and red indicate a positive correlation and negative correlation, respectively. **C**. Co-regulatory network. The co-regulatory network of *TaBBXs* was established based on the PCCs of these gene pairs using transformed qPCR data. Different colors and styles indicate the different significance levels of the co-regulated gene pairs

Several *BBXs*, such as *AtBBX19* and *AtBBX21* of the subfamily IV are involved in seed germination, suggesting that that this subfamily is likely to be involved in seed germination [25]. To explore whether *TaBBXs* have regulatory roles in wheat seed dormancy and germination, we first used quantitative expression experiments to analyze the expression of subfamily IV members in different wheat tissues (roots, stems, leaves and seeds). The expression of 14 *TaBBXs* (*TaBBX2-2A/-4–2A/-7-2B/-11-2D/-25–5A/-31-5B/-37-5D/-43–6A/-45–6A/-54-6B/-61-6D/-67–7A/-77-7B/-87-7D*) was higher in seeds than in other tissues (Fig. 3A). Subsequently, we observed the expression patterns of these 14 *TaBBXs* at 3/-4/-5 weeks after anthesis (representing dormancy acquisition, with dormant seeds as a control), 5 d/-15 d/-30 d post-ripening (representing dormancy release, with dormant seeds as a control) and 10 h imbibition (representing dormancy release, with 0 h as a control) in weak-dormancy J411 and strong-dormancy HMC21. The results indicated that seed dormancy was gradually acquired as seeds matured, and the expression levels of the 14 *TaBBXs* were up-regulated in J411 and HMC21 seeds (Fig. 4A, Fig.S10A). In contrast, seed dormancy was gradually broken at different periods of post-ripening and imbibition, and the expression of the 14 *TaBBXs* was down-regulated in J411 and HMC21 seeds (Fig. 5A A and Fig.S10B-C). Notably, the expression levels of the 14 *TaBBXs* were consistently and significantly higher in HMC21 seeds than in J411 seeds during dormancy acquisition and release, suggesting that the 14 *TaBBXs* may be involved in inducing and maintaining seed dormancy.

**Fig. 6:**
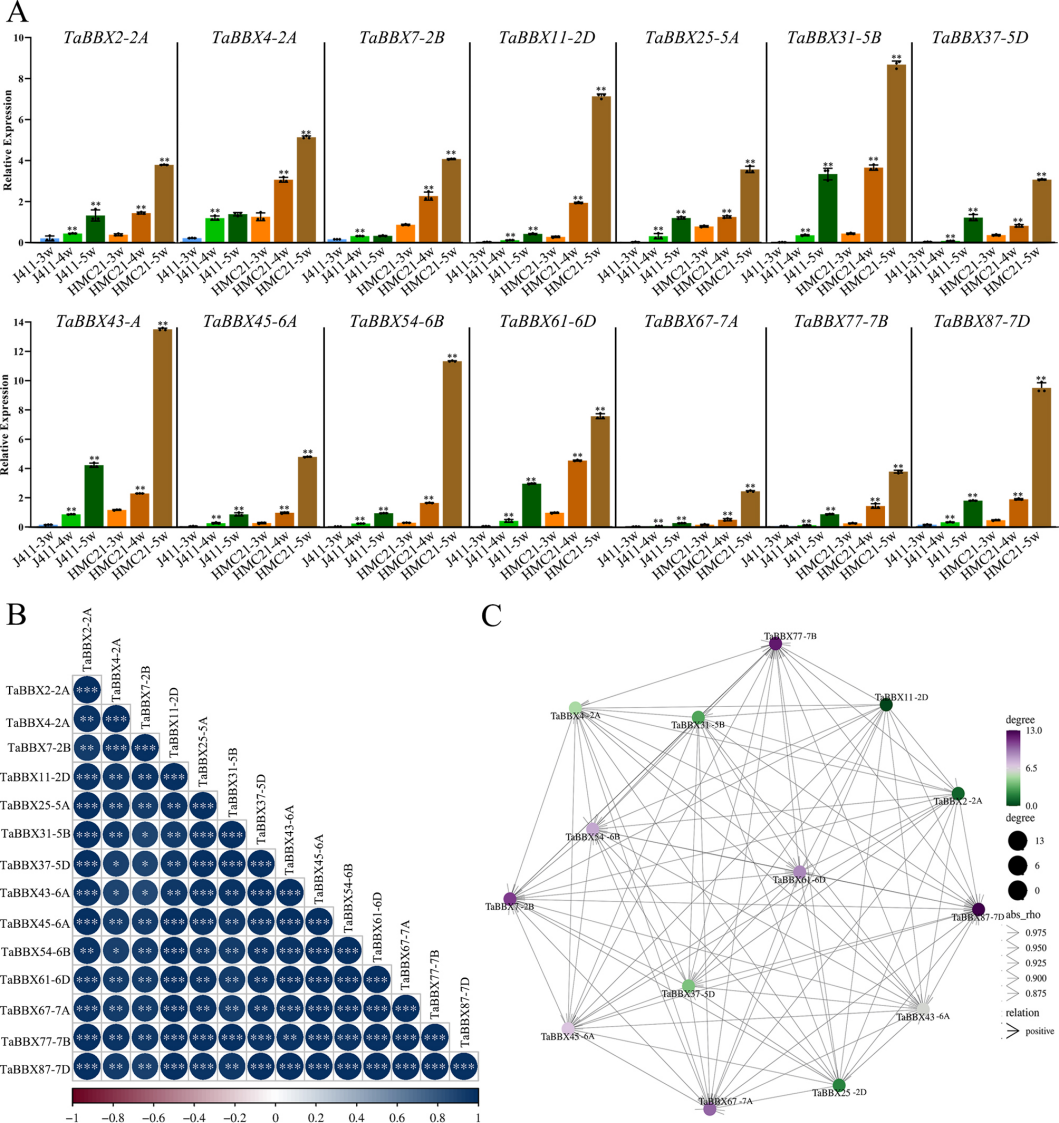
Expression patterns of *TaBBXs* at different imbibition stages by qRT-PCR. **A**. Expression patterns of *TaBBXs* after the 0 h and 10 h imbibition treatments, with 0 h as the control. Three biological replicates per organization. Abbreviations represent the seed samples of the two wheat varieties Jing 411 (J411) and Hongmangchun 21 (HMC21) collected at 0 and 10 h imbibition stages: J411-0 h, J411-0 h; J411-10 h, J411-10 h; HMC21-0 h, HMC21-0 h; HMC21-10 h, HMC21-10 h. **B**. Correlation analysis using the R package program. Each correlation is shown by the shades of blue and red and the size of the circle shape. Blue and red indicate a positive correlation and negative correlation, respectively. **C**. Co-regulatory networks. The co-regulatory networks of *TaBBXs* were established based on the PCCs of these gene pairs using transformed qPCR data. Different colors and styles indicate the different significance levels of the co-regulated gene pairs

To investigate the linkage between these genes, a co-regulatory network was constructed based on their relative gene expression levels of PCCs. A positive correlation was observed between all subfamily IV *TaBBXs* in different tissues (Figs. 3B-C, 4B-C, 5B-C and 6B-C), which also showed positive correlation in the other three treatments.

### Subcellular localization, transcriptional activation and interaction analysis of *TaBBX2-2A/-4-2A/-11-2D*

**Fig. 7:**
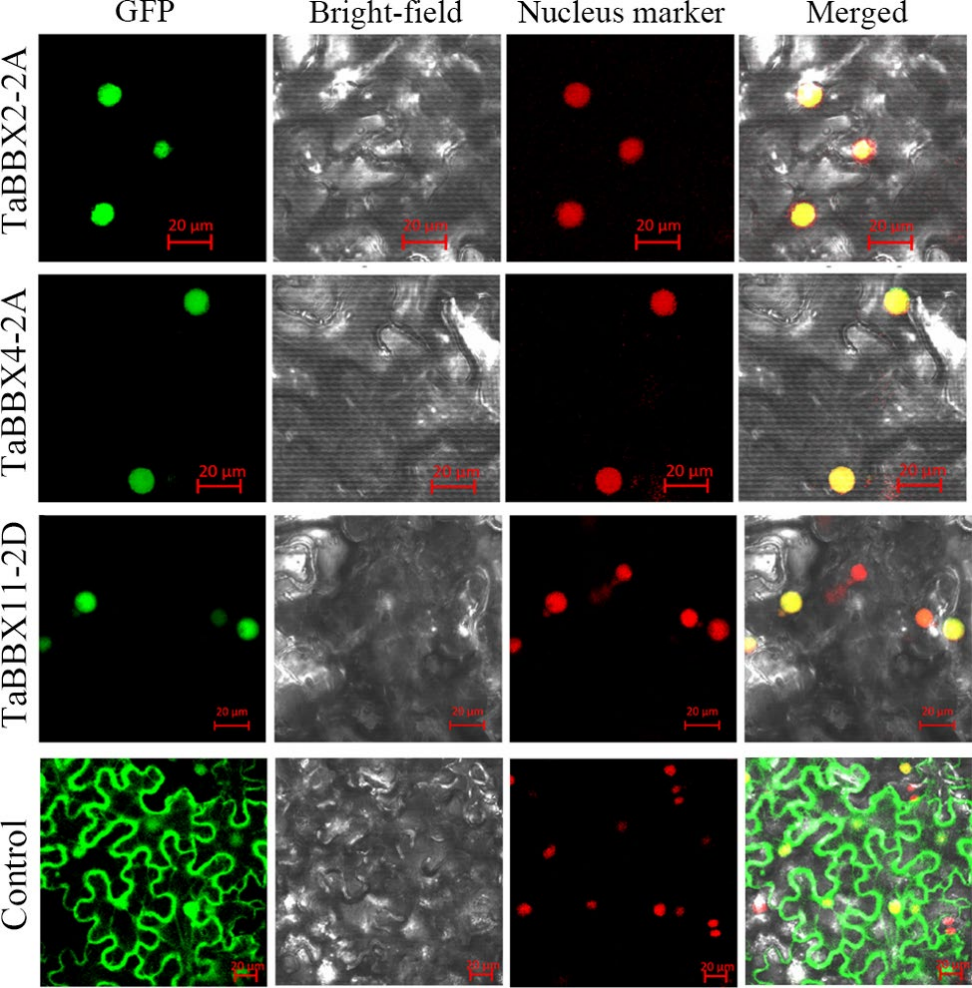
Nuclear localizations of TaBBX2-2A, TaBBX4-2A, and TaBBX11-2D. *TaBBX2-2A/-4–2A/-11-2D-GFP* construct and the control vector 1300 were transformed into Nicotiana tabacum leaves. The GFP in leaf cells were observed by confocal microscopy

Based on amino acid sequence similarity, the above 14 *TaBBXs* were found to belong to the same subfamily IV as *AtBBX19* and *AtBBX21*. Therefore, we randomly selected three candidate genes (*TaBBX2-2A*, *TaBBX4-2A* and *TaBBX11-2D*) to validate their subcellular localization. The *TaBBX2-2A-GFP*, *TaBBX4-2A-GFP*, and *TaBBX11-2D-GFP* expression vectors were transiently expressed in tobacco leaves, respectively. *TaBBX2-2A*, *TaBBX4-2A* and *TaBBX11-2D GFP* signaling was observed only in the nucleus, whereas plants transformed with the constitutively expressed *GFP* control vector showed *GFP* distributed throughout cells (Fig. 7). These findings demonstrate that TaBBX2-2A, TaBBX4-2A, and TaBBX11-2D were all nuclear proteins.

**Fig. 8:**
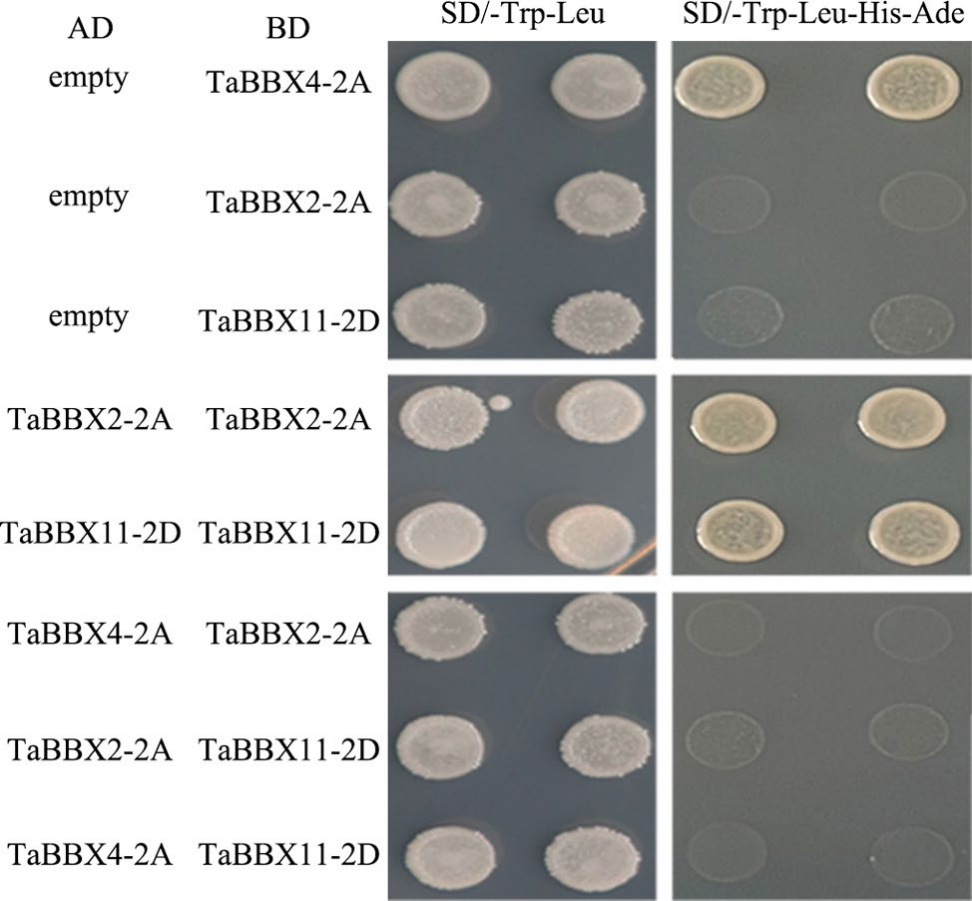
Transcriptional self-activity and interaction analysis of TaBBX2-2A, TaBBX4-2A, and TaBBX11-2D

TFs usually have transcriptional activation activity. We further transformed *TaBBX2-2A-BD*, *TaBBX4-2A-BD* and *TaBBX11-2D-BD* expression vectors into *Y2GoLD* yeast cells to assess transcriptional self-activity, respectively. *TaBBX2-2A-BD*, *TaBBX4-2A-BD* and *TaBBX11-2D-BD* grew well on SD/-Trp-Leu plates. On SD/-Trp-Leu-His-Ade plates, *TaBBX4-2A-BD* grew well, while *TaBBX2-2A-BD*, and *TaBBX11-2D-BD* failed to grow. Thus, TaBBX4-2A had transcriptional activation activity, while *TaBBX2-2A* and *TaBBX11-2D* had no transcriptional activation activity (Fig. 8).

Protein interaction is one of the main ways that BBXs perform their functions. To explore the interaction between TaBBX2-2A, TaBBX4-2A and TaBBX11-2D, we performed a yeast two-hybrid (Y2H) assay. There was no interaction between TaBBX2-2A, TaBBX4-2A, and TaBBX11-2D, but TaBBX2-2A and TaBBX11-2D interacted with each other in yeast cells (Fig. 8).

## Discussion

### Identification and phylogenetic relationship in *TaBBXs*

The *BBX* gene family is a family of zinc-finger TFs containing the B-box and CCT domains. Several members of the family have been identified in plant species, such as 32, 30, 29, 30, 64, 37 and 24 members in *Arabidopsis*, rice, tomato, potato, apple, pear and grape, respectively [21–23]. In this study, 96 *BBXs* were obtained from the wheat genome database. Compared with the above species, the higher number of *TaBBXs* in wheat may be because it is a heterohexaploid and has three partially homologous genomes, with more homologous genes. A higher homozygote retention rate may also partly explain the higher number.

Based on the number of B-box domains and the presence of the CCT domain, 96 TaBBXs were divided into five subfamilies, with the distribution of members not consistent with *Arabidopsis*, but consistent with other species (such as rice). For example, TaBBX26-5A, TaBBX27-5A and TaBBX32-5B in subfamily III had no CCT domain, and the same phenomenon was observed for SlBBX9, SlBBX11 and SlBBX12 in tomato subfamily III and OsBBX7 and OsBBX19 in rice subfamily III [2]. TaBBX17-4A, TaBBX20-4B, TaBBX21-4D, TaBBX73-7A, TaBBX82-7B, and TaBBX92-7D in subfamily II lost a BBX domain, and the same phenomenon was observed for OsBBX25 and OsBBX27 in rice subfamily I and ZmBBX7 in maize subfamily II [2]. In terms of BBX protein evolution, there are significant differences in the consistency of amino acid sequences between the B-box1 and B-box2 domains in animals. In contrast, the amino acid sequences of the B-box1 and B-box2 domains in plants, although slightly different, are topologically identical and strictly conserved. Thus, it is the diversity of C-terminal sequences of BBX proteins, such as the VP motif, which is characterized by radiation variation [28]. In the present study, 39 TaBBXs contained VP motifs, suggesting that there may be functional diversity in TaBBXs.

In addition, BBXs in most green algae have only one B-box domain, but the more primitive *Chlamydomonas reinhardtii* BBX protein CrBBX1 has two B-box domains, indicating that the original BBX protein had only one B-box domain and that it was only during later evolution that the genomic sequence containing the B-box domain underwent a duplication event, probably before green plants, and then the CCT domain was formed. Deletion of both B-box and CCT domains during subsequent evolution, and further replication events of the B-box domain helped BBX proteins to complete their evolution into different structural types [2]. Fifteen TaBBXs contained only one B-box domain, indicating that TaBBXs may have undergone multiple genome duplication events.

Gene structures diversity also plays an important role in the evolution of gene families. Our current results showed that *TaBBXs* were highly variable in length, and encoded 146 to 490 aa. *TaBBXs* on the same phylogenetic tree branch within the same group have highly similar gene structures, and the total number of introns is smaller, demonstrating evolutionary conservatism. We identified 36 homologous *BBX* pairs, including two *Ta/Ta* paralogous pairs, 33 *Ta/Os* orthologous pairs and one *Ta/At* orthologous pair. The Ka/Ks ratio of 23 pairs was > 1, suggesting that *BBXs* underwent strong positive selection during evolution. *TaBBXs* distributed on each chromosome varied. Chromosomes 1A, 1B, and 1D had no genes, chromosomes 3A, 3B, and 3D had only one *TaBBX* gene, while chromosomes 7A and 7D contained up to 10 *TaBBXs.* This biased distribution pattern was also observed in the *Arabidopsis* genome.

*TaBBXs* are primarily targeted and regulated by *miR9780* and *miR9657a*. *miR9657a* was previously reported to be associated with wheat *Fusarium head blight* (FHB), implying that four *TaBBXs* (*TaBBX35-5B/-67–7A/-77-7B/-87-7D*) may confer resistance to the disease [2]. *miR408* and *miR9780* were reported to be involved in salt stress response, suggesting that seven *TaBBXs* (*TaBBX24-5A/-30-5B/-36-5D/-42–6A/-51-6B/-60-6D/-69–7A*) may be responsive to salt stress [29, 30]. *miR397-5p* is involved in drought stress response, suggesting that the three *TaBBXs* (*TaBBX1-2A/-76–7A/-85-7B*) may be responsive to drought stress [31]. *miR399* is involved in low temperature stress response, suggesting that *TaBBX82-7B* may be responsive to low temperature stress [32]. *miR171b* was reported to be associated with rice blast and *miR171a* was also reported to be associated with wheat powdery mildew [2]. Thus, our current results suggesting that three *TaBBXs* (*TaBBX40-6A/-49-6B/-85-7B*) may be involved in wheat disease resistance. However, more experimental data is needed to confirm these speculations.

Promoter *cis*-acting elements affect gene transcription, and their type and number can lead to differential gene expression. In the present study, we found different numbers and types of *cis*-acting elements in the *TaBBXs* promoter regions. Besides light-response elements such as G-box and GT1-motif, *TaBBXs* promoter regions contained various elements involved in hormone and stress responses, such as ABRE, TGACG-motif, MBS and LTR. Thus, our results indicated that *TaBBXs* are not only involved in light signal regulation, but may also play an important role in response to some stresses.

### Identification of candidate genes associated with seed dormancy and germination

Gene expression patterns can provide important information on the gene functions. In this study, we obtained three publicly available GEO datasets to analyze the expression of *TaBBXs.* In GSE12508 data, most *TaBBXs* were differentially expressed in seeds, embryos and endosperms, such as, *TaBBX2-2A/-4–2A/-7-2B/-11-2D* were highly expressed at 3–5 DAP C, and *TaBBX43-6A/-52-6B/-61-6D* were lowly expressed at 22 DAP-EN and 3–5 DAP C. In GSE49821 data, most *TaBBXs* were differentially expressed in JM20 seeds at different germination stages, such as, *TaBBX2-2A/-4–2A/-7-2B/-9-2B/-11-2D/-17–4A/-20-4B/-22-4D/-40–6A/-47–6A/-49-6B/-56-6B/-71–7A/-72–7A/-80-7B/-81-7B/-90-7D* were lowly expressed with increasing imbibition time (dormancy release). In GSE118474 data, most *TaBBXs* were also differentially expressed in the embryos and endosperms at different seed development stages (dormancy acquisition) in Zhou8425B, such as, *TaBBX1-2A/-3–2A/-8-2B/-17–4A/-21-4D/-30-5B/-36-5D/-55-6B/-64-6D* were more highly expressed in M EB and M EN than in G EB and G EN. These results indicated that *TaBBXs* may play regulatory roles in wheat seed dormancy and germination.

Several *BBXs* are involved in seed germination. Xu et al. (2014) showed that BBX21 negatively regulated ABI5 expression by interfering with HY5 binding to the ABI5 promoter, thereby promoting *Arabidopsis* seed germination [4]. Bai et al. (2019) found that AtBBX19 inhibited seed germination through induction of *ABI5* [27]. However, there is no report on the role of *TaBBXs* in seed dormancy and germination. Based on the similarity of amino acid sequences and the results of expression profiling, some *TaBBXs* in subfamily IV may be related to seed germination. Therefore, we first subjected 29 *TaBBXs* belonging to the same subfamily IV as *AtBBX19* and *AtBBX21* to qRT-PCR firstly in different wheat tissues (roots, stems, leaves and seeds). The expression of 14 *TaBBXs* (*TaBBX2-2A/-4–2A/-7-2B/-11-2D/-25–5A/-31-5B/-37-5D/-43–6A/-45–6A/-54-6B/-61-6D/-67–7A/-77-7B/-87-7D*) was higher in seeds than in other tissues. Next, we detected the expression levels of 14 *TaBBXs* in two wheat varieties with contrasting seed dormancy and germination (J411 and HMC21). The expression of 14 *TaBBXs* was increased during seed dormancy acquisition and was down-regulated during dormancy release. In addition, the expression levels of 14 *TaBBXs* were higher in HMC21 with strong dormancy than in J411 with weak dormancy. These results suggest that these 14 *TaBBXs* positively regulated seed dormancy and negatively mediated germination. To investigate the relationships between *TaBBXs* under different conditions, co-regulatory networks were constructed based on PCCs of GEO data or relative gene expression levels, and a series of *TaBBXs* showed significant correlation of expression changes in the co-regulatory network. These results suggest that *TaBBX2-2A/-4–2A/-7-2B/-11-2D/-25–5A/-31-5B/-37-5D/-43–6A/-45–6A/-54-6B/-61-6D/-67–7A/-77-7B/-87-7D* shared common positive correlation under different treatments.

*BBXs*, as important TFs, primarily function by transcriptional regulation and protein interaction. For example, AtBBX21 not only interacted with AtBBX22, but also bound directly to the promoter region of the *HY5* gene, thereby enhancing its expression [33]. In the present study, we randomly selected three candidate genes (*TaBBX2-2A*, *TaBBX4-2A* and *TaBBX11-2D*) from the above 14 *TaBBXs* for further research. Nuclear localization and transcriptional activite are the characteristic functions of TFs. To further explore the functions of *TaBBX2-2A*, *TaBBX4-2A* and *TaBBX11-2D*, we first performed subcellular localization experiments. TaBBX2-2A, TaBBX4-2A and TaBBX11-2D were all located in the nucleus, consistent with the prediction results. Next, we performed transcriptional activity experiments, and found that TaBBX2-2A and TaBBX11-2D were not transcriptionally active, and TaBBX4-2A had transcriptional activation activity, indicating that TaBBX2-2A and TaBBX11-2D may function by protein interaction, and TaBBX4-2A may function by transcriptional regulation. Therefore, Y2H experiments were performed, and the results showed that TaBBX2-2A and TaBBX11-2D interacted with each other, suggesting that they may regulate seed dormancy and germination by self-interaction.

## Conclusion

To summarize, we identified 96 *TaBBXs* in five subfamilies (I-V) from the wheat genome, of which 39 *TaBBXs* contained VP motifs at the C-terminus. By analyzing gene structures and expression patterns, 14 *TaBBXs* were possibly associated with seed dormancy and germination, and their interaction networks were constructed. Particularly, the nuclear localization, transcriptional activity, and protein interaction characteristics of TaBBX2-2A, TaBBX4-2A and TaBBX11-2D were further investigated. Our results provide valuable information for further studies on *BBXs*, elucidating not only their functions in seed dormancy and germination, but also their roles in stress response.

## Materials and methods

### Materials and treatment

The experimental materials were two wheat varieties (Jing 411, J411; Hongmangchun 21, HMC21) with extreme differences in dormancy determined in our previous research [26]. J411 has a low level of seed dormancy, while HMC21 exhibits a high level of seed dormancy. Mature dormant seeds were selected for the 0 h/4 h/6 h/10 h imbibition treatments, with 0 h as the control. In addition, tissues of field grown wheat were collected from April to July, including wheat seeds at the seed development stage (25 d/30 d/35 d post anthesis), wheat seeds at the post-maturity stage (5 d/15 d/30 d post-harvest), and the roots, stems, leaves and other tissues of wheat at the growth stage. Three replicates of each sample were used. All samples were frozen in liquid nitrogen and stored at -80 °C.

### Identification of *TaBBX* gene members

Wheat whole genome sequences, CDS sequences, protein sequences and annotation files were downloaded from the Ensembl Plants database [26]. *Arabidopsis* and rice B-box protein sequences were downloaded from The *Arabidopsis* Information Resource (http://www.arabidopsis.org) and Rice Genome Annotation Project database (http://rice.plantbiology.msu.edu/), respectively. *TaBBX* candidate genes were obtained using the Pfam database (http://pfam.janelia.org/search/sequence), and then verified for the presence of specific B-box domains using SMART (http://smart.embl-heidelberg.de/), and NCBI online tool CDD (http://www.ncbi.nlm.nih.gov/Structure/cdd/wrpsb.cgi). *TaBBX* amino acid sequences were analyzed for isoelectric point (pI), aliphatic index (Ai), etc. using the ExPASy website (http://expasy.org/) [26]. ClustalX 2.11 was used for *TaBBXs* multiple sequence alignment [26].

### Phylogenetic analysis

Phylogenetic trees were constructed using the maximum likelihood method in MEGA7.0 software. The substitution model was calculated with ModelFinder (best-fit model: JTT + G5 chosen by the optimal model was quizzed based on the data; bootstrap: 1000) [34, 35]. The wheat *BBX* gene family was classified based on the results of *Arabidopsis* and rice *BBX* gene family classification [21]. Paralogous and orthologous pairs were determined according to previously described research [34]. Ka, Ks and Ka/Ks values for homologous pairs were obtained according to the algorithm by Cheng et al. (2021) [34]. Three homologous groups (A:B:D, triplets) were analyzed according to the method of Cheng et al. (2021) [34, 35].

### Structure analysis

*TaBBX* domains were analyzed with the NCBI online tool CDD. Chromosomal location and gene structure information of *TaBBXs* was extracted from the wheat genome database GFF3 files and visualized using MapGene2 Chromosome version 2.0. (http://mg2c.iask.in/mg2c_v2.0/) [34] and GSDS tools (http://gsds.cbi.pku.edu.cn/) [26] respectively. A single gene cluster comprised two or more genes separated by less than 200 Kb on a single chromosome [26]. Subcellular localization, signal peptide and targeted miRNAs prediction were performed by WoLF PSORT, SignalP 4.1 and PsRNATarget, respectively [34].

### Promoter and gene ontology (GO) analysis

Each *TaBBX* gene promoter region was extracted at 1500 bp from the wheat genome, and analysis of the type, number, and functions of cis-acting elements was performed using the PlantCARE database (http://bioinformatics.psb.ugent.be/webtools/plantcare/) [26]. GO annotation for each *TaBBX* was extracted from the Wheat Whole Genome Database [26].

### RNA extraction and real-time fluorescence quantitative PCR analysis

The *TaBBX* gene expression data in accession numbers GSE12508, GSE49821 and GSE118474 were obtained from NCBI Gene Expression Omnibus (https://www.ncbi.nlm.nih.gov/geo/) and NetAffx Analysis Center (http://www.affymetrix.com/), and visualized using the Heatmapper Plus tool (http://bar.utoronto.ca/ntools/cgi-bin/ntools thermal mapper) [34]. GSE12508 comprises gene expression analysis data during ‘Chinese Spring’ development. GSE49821 contains gene expression during seed germination of wheat cultivar Jimai 20 (JM20). GSE118474 has gene expression analysis data during grain embryo and endosperm development in wheat cultivar Zhou 8425B.

Total wheat RNA was extracted using the RNAprep Pure Plant Kit as a template, and cDNA was reverse transcribed using the PrimeScript RT Reagent Kit with gDNA Eraser (Perfect Real Time) (TaKaRa). The *TaBBX* primers were designed using Primer Premier 6 software (Table S13), and *TaActin1/-2* was used as the internal reference gene [26, 36]. The PCR system consisted of cDNA 2 µL, 2×SYBR Premix ExTaq 10 µL, 50×ROX Reference Dye 0.4 µL, F/R primers 0.8 µL, and ddH_2_O 6 µL. The amplification program was 94 °C for 30 s, and 40 cycles of 94 °C for 5 s, 58 °C for 15 s, 72 °C for 10 s. Three biological replicates were performed for each treatment, and the relative gene expression was calculated using the 2^−ΔΔCT^ method.

### Subcellular localization

The coding regions of *TaBBX2-2A*, *TaBBX4-2A*, and *TaBBX11-2D* were cloned in J411 and HMC21 by PCR (Table S13). The CDS of *TaBBX2-2A*, *TaBBX4-2A*, and *TaBBX11-2D* was inserted into the *pCambia1300* vector (digested with *Sal*I and *BamH*I) between the cauliflower mosaic virus *35 S* promoter and the green fluorescent protein (*GFP*) sequence to form translational fusions with the N-terminus of *GFP*. The suspension containing the recombinant plasmid and nuclear marker with RFP (OsART1) was injected into the lower epidermis of tobacco leaves and labeled. *GFP* fluorescence was observed under a confocal laser-scanning microscope (Leica TCS SP5; Leica Microsystems, Wetzlar, Germany).

### Yeast two hybrid assay

The CDS of *TaBBX2-2A*, *TaBBX4-2A*, and *TaBBX11-2D* was inserted into the *pGBKT7* (*BD*) and *pGADT7* (*AD*) vectors and the target fragments were digested with the *EcoR*I and *BamH*I restriction enzymes to generate recombinant plasmid vectors, respectively (Table S13). The constructed vector plasmids were transformed into *Y2HGold* yeast receptor cells. The experimental groups were plated on SD/-Leu/-Trp and SD/-Ade/-Leu/-Trp/-His media and incubated at 30 °C for 3-5d to verify transcriptional self-activity and protein interactions.

### Statistical analysis

Microsoft Excel and GraphPad 5 software were used for data processing and visualization [34]. Significance was analyzed performed using SPSS software, with *P* < 0.05 indicating significant difference and *P* < 0.01 indicating highly significant differences. Pearson correlation coefficients (PCCs) and *P*-values of the *TaBBX* gene expression levels were calculated and visualized using the R package [34]. * and ** denote correlations with *P* ≤ 0.05 and *P* ≤ 0.01, respectively.

### Electronic supplementary material

Below is the link to the electronic supplementary material.

Supplementary Material 1

Supplementary Material 2

Supplementary Material 3

Supplementary Material 4

Supplementary Material 5

Supplementary Material 6

Supplementary Material 7

Supplementary Material 8

Supplementary Material 9

Supplementary Material 10

Supplementary Material 11

Supplementary Material 12

Supplementary Material 13

Supplementary Material 14

## Data Availability

Data from this article were obtained from the Ensembl database (http://plants.ensembl.org/index.html). The genome sequences of wheat, rice and Arabidopsis were downloaded from from Ensembl database (http://plants.ensembl.org/index.html), Rice Genome Annotation Project database (http://rice.plantbiology.msu.edu/) and PlantTFDB (http://planttfdb.cbi.pku.edu.cn).

## Abbreviations

BBX: B-box
CCT: Conserved carboxy- terminal
VP: valine-proline
qRT-PCR: quantitative real-time PCR
Ks: number of synonymous substitutions per synonymous site
Ka: number of non-synonymous substitutions per non-synonymous site
NJ: neighbor-joining
CDS: coding sequence
Bp: base pair
aa: amino acids
MW: molecular weight
pI: isoelectric point
Da: Dalton
PHS: Pre-harvest sprouting
GRAVY: Grand average of hydropathicity
AI: Aliphatic index
II: Instability index
G-EB: 14 d Embryo
M-EB: 25 d Embryo
G-ES: 14 d Endosperm
M-ES: 25 d Endosperm
GSC: germinating seed coleoptile
GSR: germinating seed root
GSE: germinating seed embryo
SR: seedling root
SC: seedling crown
SL: seedling leaf
II: immature inflorescence
Fba: floral bracts before anthesis
Pba: pistil before anthesis
Aba: Anthers before anthesis
3–5 DAP C: 3–5 DAP caryopsis
22 DAP EN: 22 DAP endosperm
22 DAP EM: 22 DAP embryo
