# Supplementary material for: In silico analysis of the wheat BBX gene family and identification of candidate genes for seed dormancy and germination
Source: BMC Plant Biol. 2024 Apr 25;24:334. doi: 10.1186/s12870-024-04977-x (PMC11044412; doi:10.1186/s12870-024-04977-x)
Supplement: Supplementary file 1 — Supplementary Material 1 [file 12870_2024_4977_MOESM1_ESM.docx]

Table S1 Primers for *TaBBXs*

qRT-PCR

| Name | Primers |
| --- | --- |
| TaActin1 | F: CTTGTATGCCAGCGGTCGAAC |
|  | R: CTCATAATCAAGGGCCACG |
| TaActin2 | F: GACCGTATGAGCAAGGAGAT |
|  | R: CAATCGCTGGACCTGACTC |
| TaBBX2-2A | F: GACCTGTTCCACGACGACCAG |
|  | R: CACCCTCGCCTTCTTGCTCTG |
| TaBBX4-2A | F: GGCAGCGTCTTGGTCAGGTAC |
|  | R: CCTGGTCGTCATACTCGTGGAAC |
| TaBBX5-2A | F: CGTCGGGTCAGGAGGAGGAG |
|  | R: GGCAGCGTCTTGGTCAGGTAC |
| TaBBX7-2B | F: AGATCCACGCCGCCAACAAG |
|  | R: CCTGTCGTCCACGCAGAAGAC |
| TaBBX9-2B | F: AGGAGGAGGCAGAAGGGAACAG |
|  | R: CGGGCAGCGTCTTGGTTAGG |
| TaBBX11-2D | F: GACCTGTTCCACGACGACCAG |
|  | R: CACCCTCGCCTTCTTGCTCTG |
| TaBBX13-2D | F: CGTCGGGTCAGGAGGAGGAG |
|  | R: GGCAGCGTCTTCGTCAGGTAC |
| TaBBX14-3A | F: CACCGCCTACCCCTCCTCTC |
|  | R: GGTCCTCCACGCAGAAGAAGTAG |
| TaBBX15-3B | F: CGATGTGCCACAGGAAGAGGAG |
|  | R: GTACCGCTGCCGAACTGCTC |
| TaBBX16-3D | F: CCTACTTCTTCTGCGTGGAGGAC |
|  | R: AGACGAAGGTGTTGGCAGTGTG |
| TaBBX25-5A | F: ACCACCACCACAATGCCTATGATC |
|  | R: AGTCTGGGAACTTGAACCTTGCC |
| TaBBX31-5B | F: ACCACCACCACAATGCCTATGATC |
|  | R: AGTCTGGGAACTTGAACCTTGCC |
| TaBBX37-5D | F: ACCACCACCACAATGCCTATGATC |
|  | R: AGTCTGGGAACTTGAACCTTGCC |
| TaBBX40-6A | F: CGGAGGAGGAGAAGACGACATTG |
|  | R: GGTTCAGAGAAGCCAGGGTAGC |
| TaBBX43-6A | F: CTCGCCAGCAAGCACCAGAG |
|  | R: CGGTCCTCGACGCAGAAGATG |
| TaBBX45-6A | F: GCAGCAGCATCTCCGAGTACC |
|  | R: CCTGATACGATCCGCCTGAAGAG |
| TaBBX49-6B | F: CTGAGAACTTCGCCATTGCCAAC |
|  | R: ATCCATGCCAACGCCATCCTC |
| TaBBX52-6B | F: GACTCGGCTCTCGGCTTCAAG |
|  | R: CTGTAGTAGGCAGCGTCGTCTG |
| TaBBX54-6B | F: GGCAGCAGCATCTCCGAGTAC |
|  | R: GGTACGATCCGCCTGATGAGAAG |
| TaBBX58-6D | F: CGGAGGAGGAGAAGATGACATTGG |
|  | R: GGTTCAGAGAAGCCAGGGTAGC |
| TaBBX61-6D | F: AGATCCACGCCGCCAACAAG |
|  | R: CGGTCCTCGACGCAGAAGATG |
| TaBBX63-6D | F: GGCAGCAGCATCTCCGAGTAC |
|  | R: GGTACGATCCGCCTGAAGAGAAG |
| TaBBX67-7A | F: AGAGCCTCCACCTAAGTACCTGTC |
|  | R: CTGACCCATTGCCGCCTGAG |
| TaBBX74-7A | F: GAGGAGGAGGAGGTGGAGGAAG |
|  | R: GGCGTCGTCGAAGAGGAGTTC |
| TaBBX77-7B | F: AGAGCCTCCACCTAAGTACCTGTC |
|  | R: ACCCGTTGCCACCTGAGAATG |
| TaBBX83-7B | F: GGAGGAGGAGGAGGAGGAAGC |
|  | R: GGCGTCGTCGAAGAGGAGTTC |
| TaBBX87-7D | F: AGAGCCTCCACCTAAGTACCTGTC |
|  | R: GTCTGACCCATTACCGCCTGAG |
| TaBBX93-7D | F: GGAGGAGGAGGAGGAGGAAGC |
|  | R: GGCGTCGTCGAAGAGGAGTTC |

PCR

| S-TaBBX2-2A | F: CGGAGCTAGCTCTAGAATGAGGATCCAGTGCGACGC |
| --- | --- |
|  | R: TGCTCACCATGGATCCTCCAAGATCGGGAACGAGGA |
| S-TaBBX4-2A | F: CGGAGCTAGCTCTAGAATGAGGATCCAGTGCGACGC |
|  | R: TGCTCACCATGGATCCCCAAGATCAGGAACGAGGAG |
| S-TaBBX11-2D | F: CGGAGCTAGCTCTAGAATGAGGATCCAGTGCGACGC |
|  | R: TGCTCACCATGGATCCTCCAAGATCGGGAACGATGA |
| T-TaBBX2-2A | F: ATGGCCATGGAGGCCGAATTCATGAGGATCCAGTGCGACGC |
|  | R: GCCCTTGCTCACCATGGATCCTCATCCAAGATCGGGAACGA |
| T-TaBBX4-2A | F: ATGGCCATGGAGGCCGAATTCATGAGGATCCAGTGCGACGC |
|  | R: GCCCTTGCTCACCATGGATCCTCATCCAAGATCAGGAACGA |
| T-TaBBX11-2D | F: ATGGCCATGGAGGCCGAATTCATGAGGATCCAGTGCGACGC |
|  | R: GCCCTTGCTCACCATGGATCCTCATCCAAGATCGGGAACGA |
| AD-TaBBX2-2A | F: GGAGGCCAGTGAATTCATGAGGATCCAGTGCGACGC |
|  | R: CGAGCTCGATGGATCCTCATCCAAGATCGGGAACGA |
| AD-TaBBX4-2A | F: GGAGGCCAGTGAATTCATGAGGATCCAGTGCGACGC |
|  | R: CGAGCTCGATGGATCCTCATCCAAGATCAGGAACGA |
| AD-TaBBX11-2D | F: GGAGGCCAGTGAATTCATGAGGATCCAGTGCGACGC |
|  | R: CGAGCTCGATGGATCCTCATCCAAGATCGGGAACGA |
| BD-TaBBX2-2A | F: CATGGAGGCCGAATTCATGAGGATCCAGTGCGACGC |
|  | R: GCAGGTCGACGGATCCTCATCCAAGATCGGGAACGA |
| BD-TaBBX4-2A | F: CATGGAGGCCGAATTCATGAGGATCCAGTGCGACGC |
|  | R: GCAGGTCGACGGATCCTCATCCAAGATCAGGAACGA |
| BD-TaBBX11-2D | F: CATGGAGGCCGAATTCATGAGGATCCAGTGCGACGC |
|  | R: GCAGGTCGACGGATCCTCATCCAAGATCGGGAACGA |
