# Supplementary material for: In silico analysis of the wheat BBX gene family and identification of candidate genes for seed dormancy and germination
Source: BMC Plant Biol. 2024 Apr 25;24:334. doi: 10.1186/s12870-024-04977-x (PMC11044412; doi:10.1186/s12870-024-04977-x)
Supplement: Supplementary file 14 — Supplementary Material 14 [file 12870_2024_4977_MOESM14_ESM.docx]

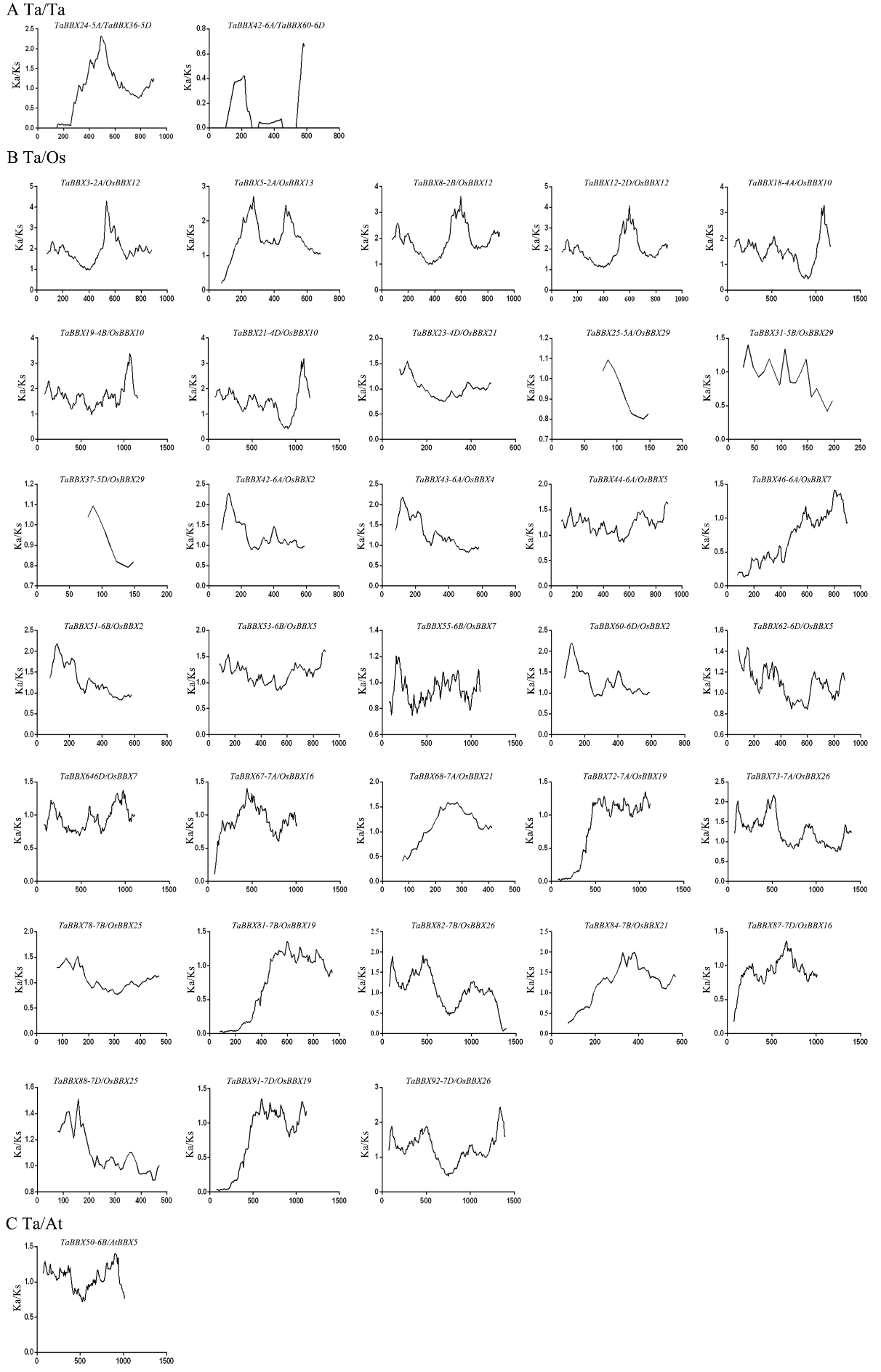


Fig.S1 Sliding window plots of the *BBXs.*

A. Sliding window plots of the *BBXs* in wheat.

B. Sliding window plots of the *BBXs* in wheat and rice.

C. Sliding window plots of the *BBXs* in wheat and Arabidopsis.


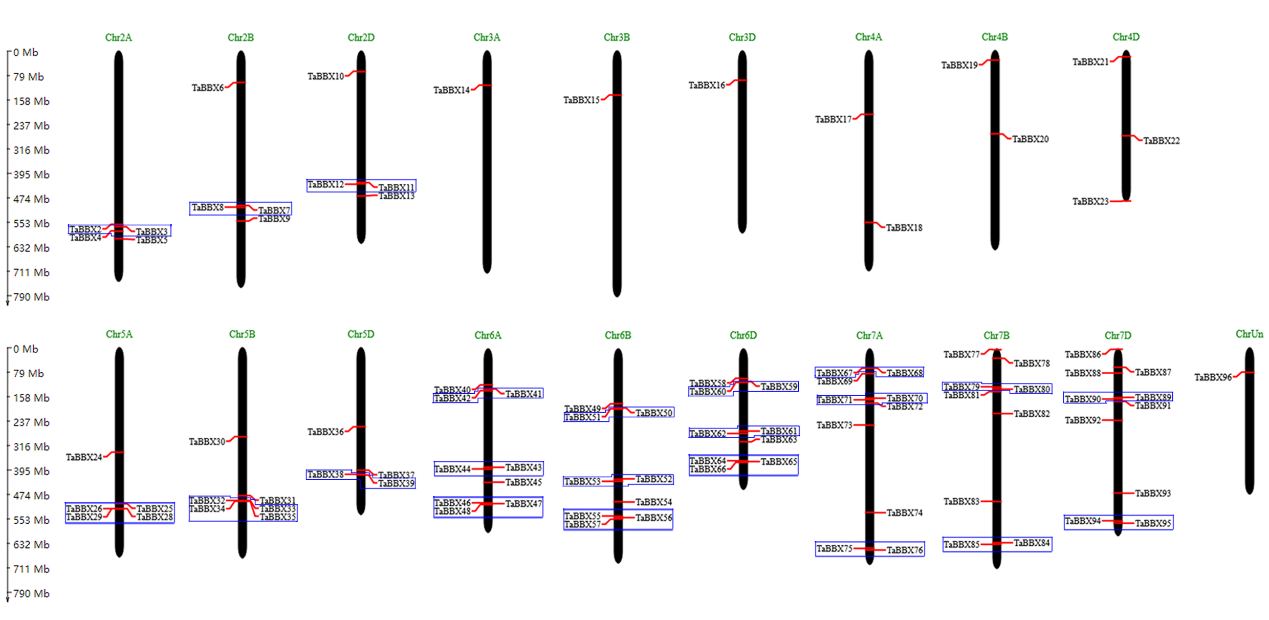


Fig.S2 Chromosomal localization of *TaBBXs.*

Respective chromosome numbers are indicated above each bar. Single gene cluster were obtained and mark it with blue b


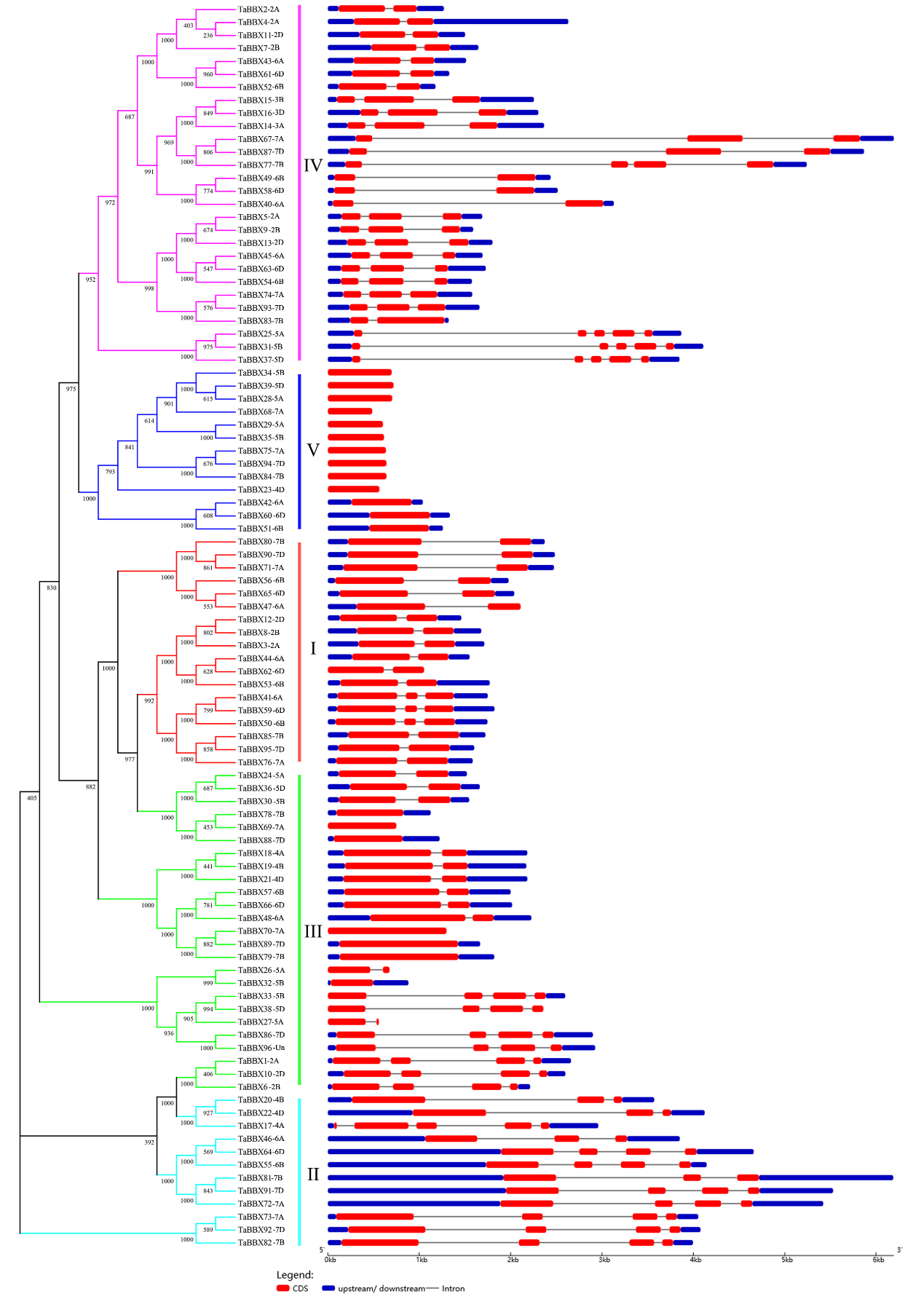


Fig.S3 Phylogenetic relationships and gene structures of *TaBBXs.*

The 96 *TaBBXs* were clustered into five subfamilies. The tree was generated using MEGA7.0 software using the neighbor-joining (NJ) method. Exons, introns, and untranslated regions (UTRs) were indicated by red rectangles, gray lines, and blue rectangles, respectively. Colored boxes indicate the subfamily based on the phylogenetic analysis.


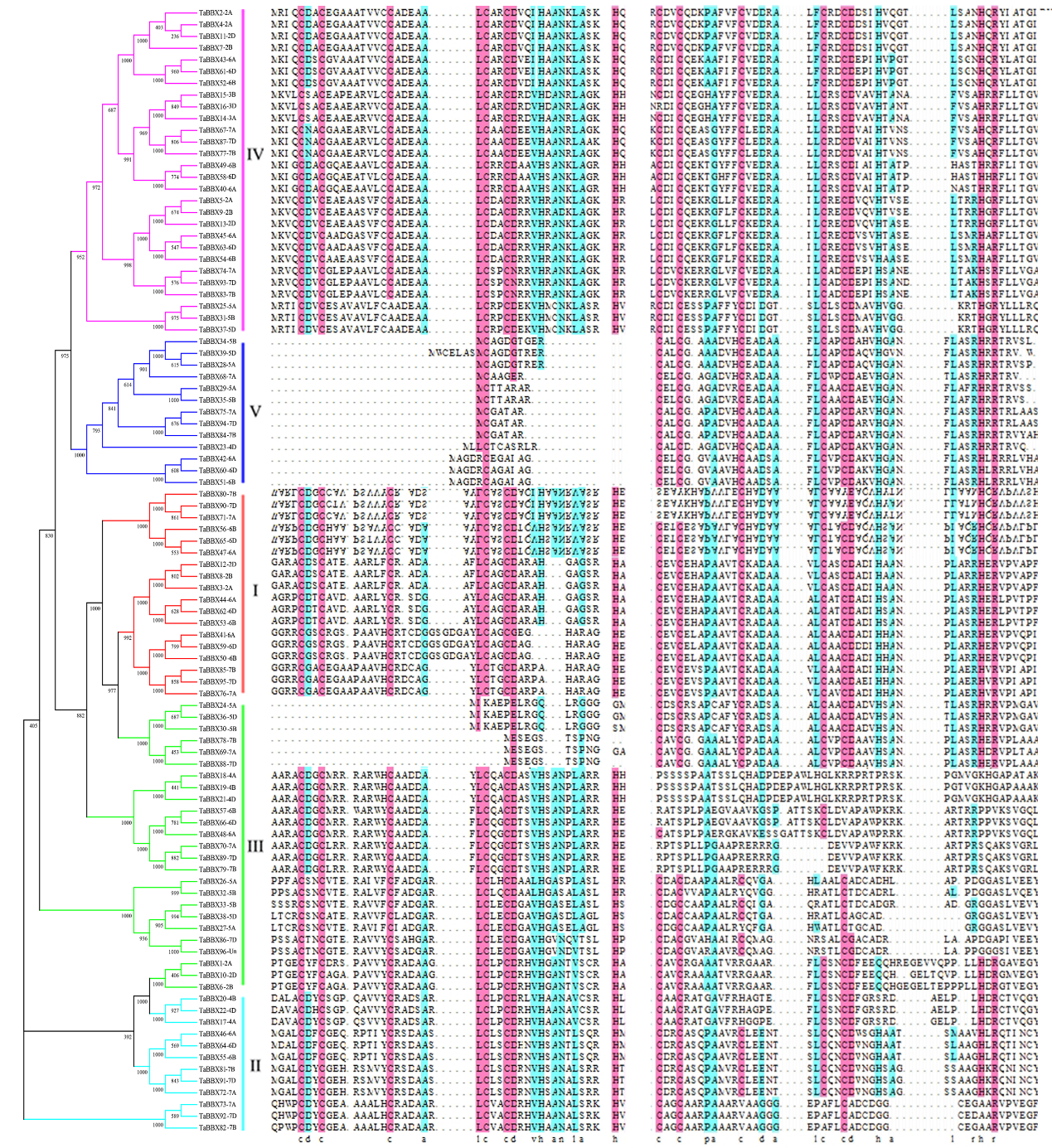


Fig.S4 Multiple sequence alignment of *TaBBXs*.


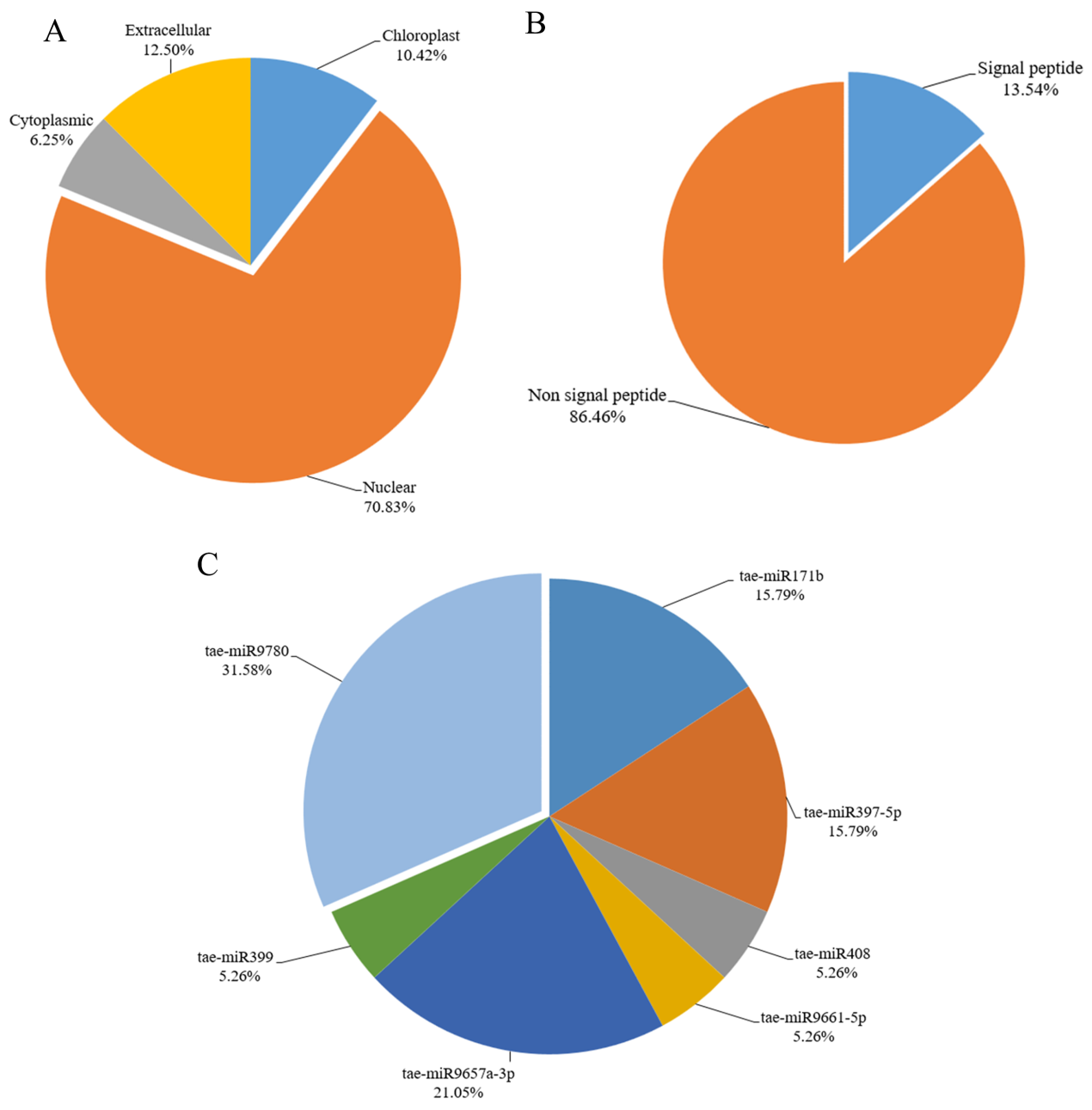


Fig.S5 Gene structures analysis of the *TaBBXs.*

A. Subcellular localization prediction of the *TaBBXs.* Different colors represent different positions.

B. Signal peptide analysis of the *TaBBXs.* Different colors represent different positions.

C. miRNA analysis of the *TaBBXs.* Different colors represent different positions.

.


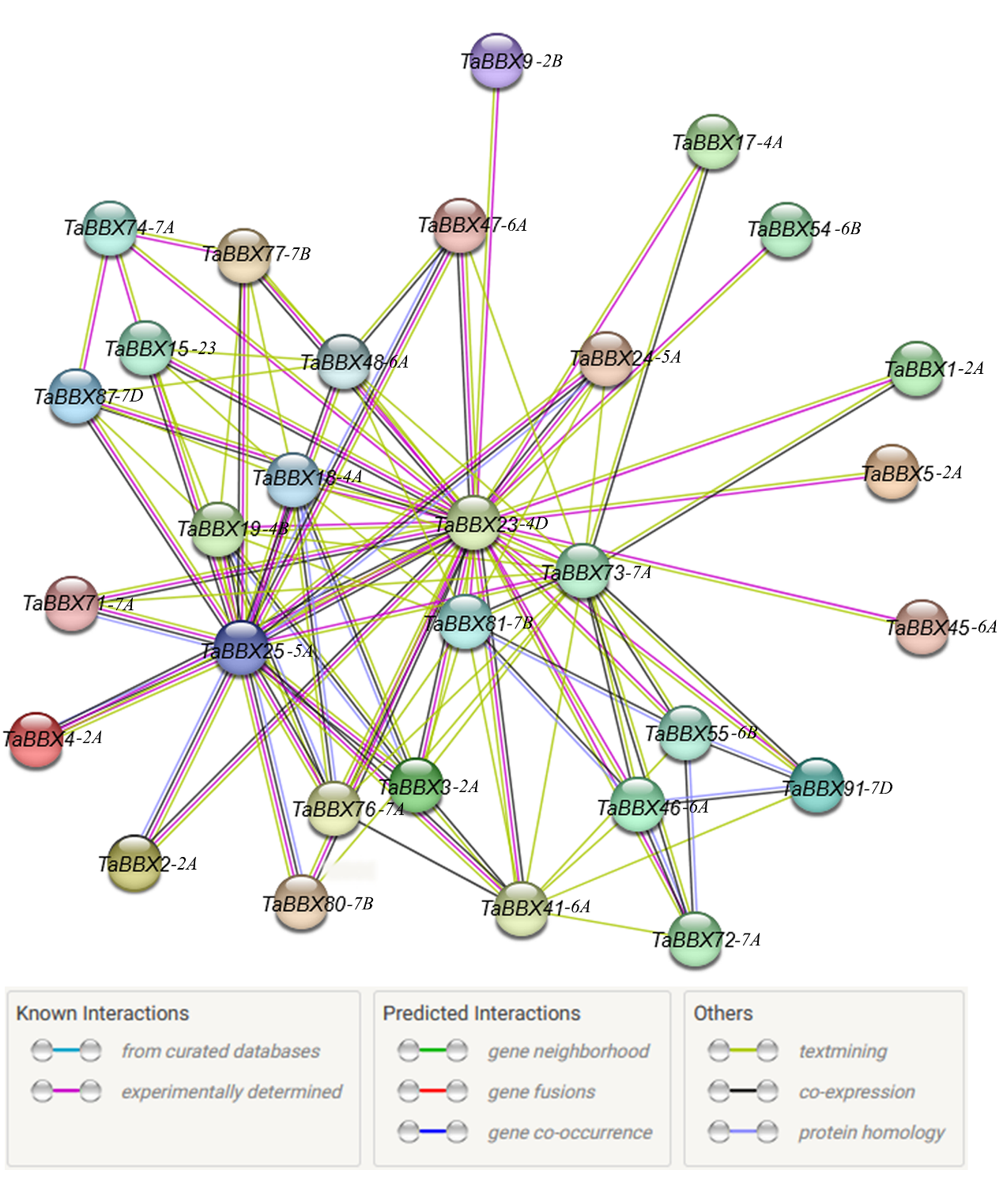


Fig.S6 Network of interactions between *TaBBXs*.

Network nodes represent proteins, and edges represent protein–protein associations. The color of each line indicates the evidence used as the basis for the predicted interaction. The minimum required interaction score was set to medium confidence (0.400).


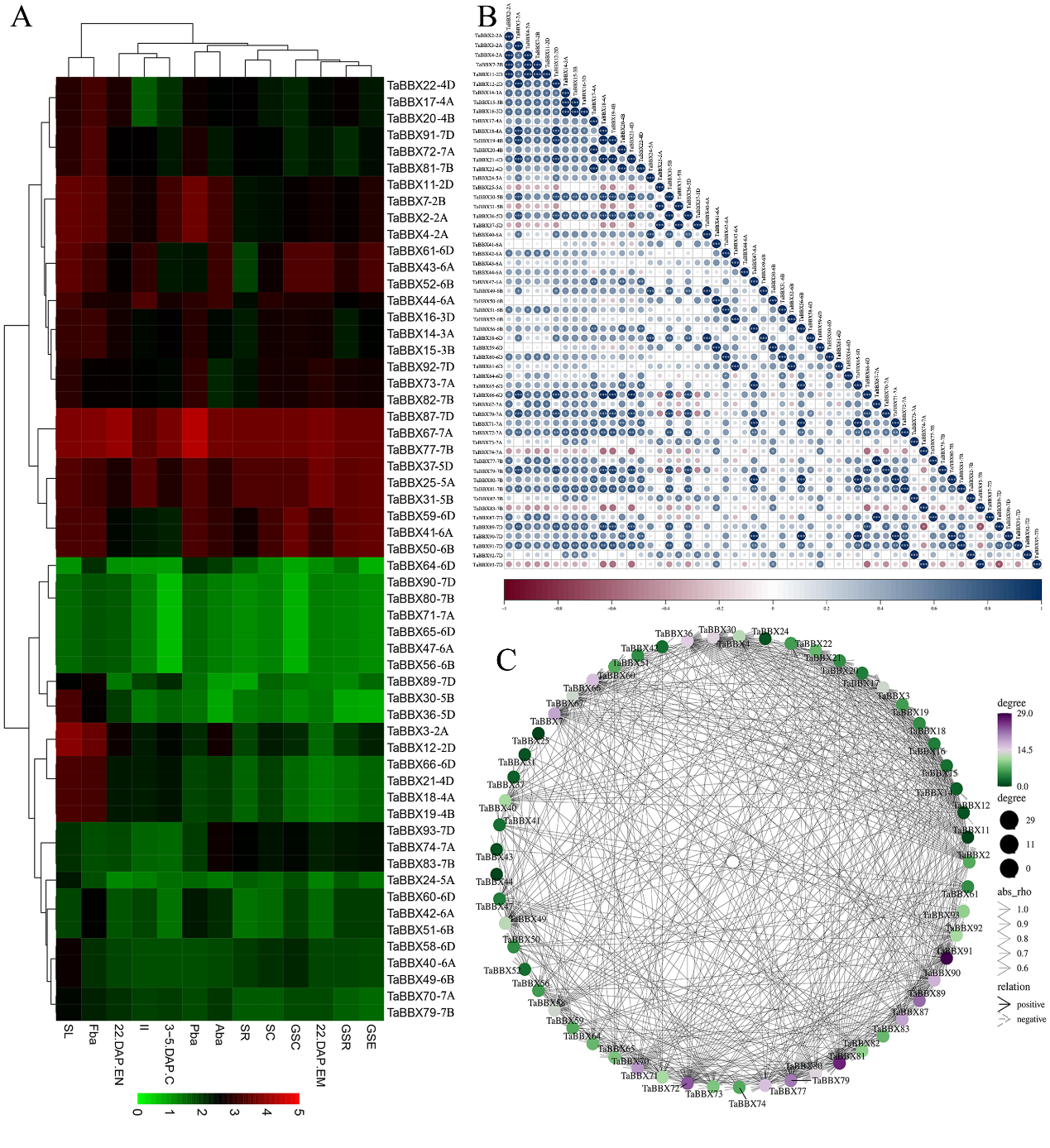


Fig.S7 Expression patterns of *TaBBXs* among GEO database.

A. Heatmap shows the hierarchical clustering of *TaBBXs* among GSE12508. Abbreviations represent specific developmental stages: GSC, germinating seed, coleoptile; GSR, germinating seed, root; GSE, germinating seed, embryo; SR, seedling, root; SC, seedling, crown; SL, seedling, leaf; II, immature inflorescence; Fba, floral bracts, before anthesis; Pba, pistil, before anthesis; Aba, anthers, before anthesis; 3-5 DAP C, 3-5 DAP caryopsis; 22 DAP EM, 22 DAP embryo; 22 DAP EN, 22 DAP endosperm.

B. Correlation analysis using the R package program. Each correlation is shown by the shades of blue and red and the size of the circle shape. Blue and red indicate a positive correlation and negative correlation, respectively.

C. Co-regulatory networks. The co-regulatory networks of *TaBBXs* were established based on the PCCs of these gene pairs using transformed qPCR data. Different colors and styles indicate the different significance levels of the co-regulated gene pairs.


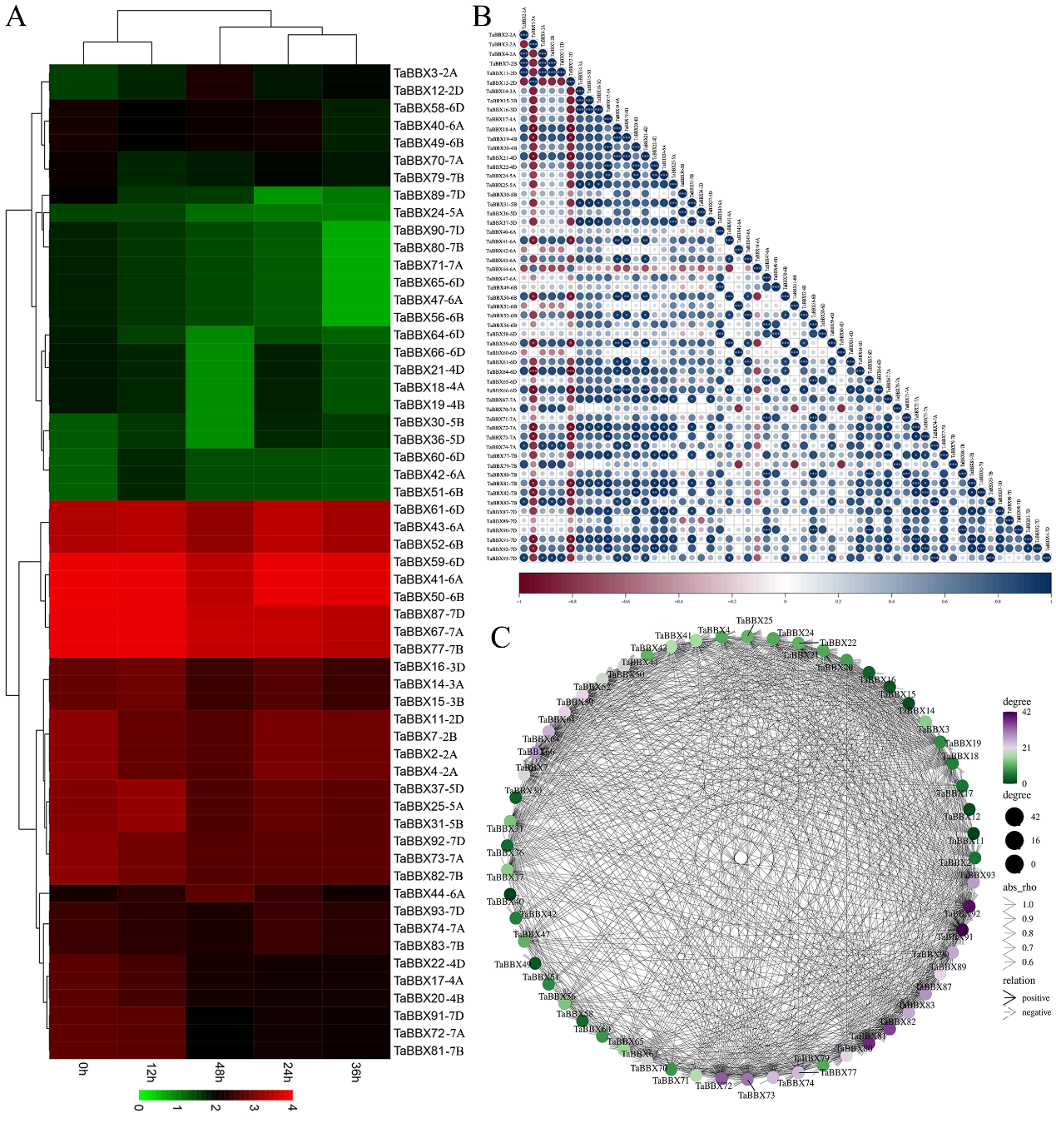


Fig.S8. Expression patterns of *TaBBXs* among GEO database.

A. Heatmap shows the hierarchical clustering of *TaBBXs* among GSE49821. Abbreviations represent Jimai 20 (JM20) germinating seeds imbibition time: 0, 12, 24, 36 and 48 hour.

B. Correlation analysis using the R package program. Each correlation is shown by the shades of blue and red and the size of the circle shape. Blue and red indicate a positive correlation and negative correlation, respectively.

C. Co-regulatory networks. The co-regulatory networks of *TaBBXs* were established based on the PCCs of these gene pairs using transformed qPCR data. Different colors and styles indicate the different significance levels of the co-regulated gene pairs.


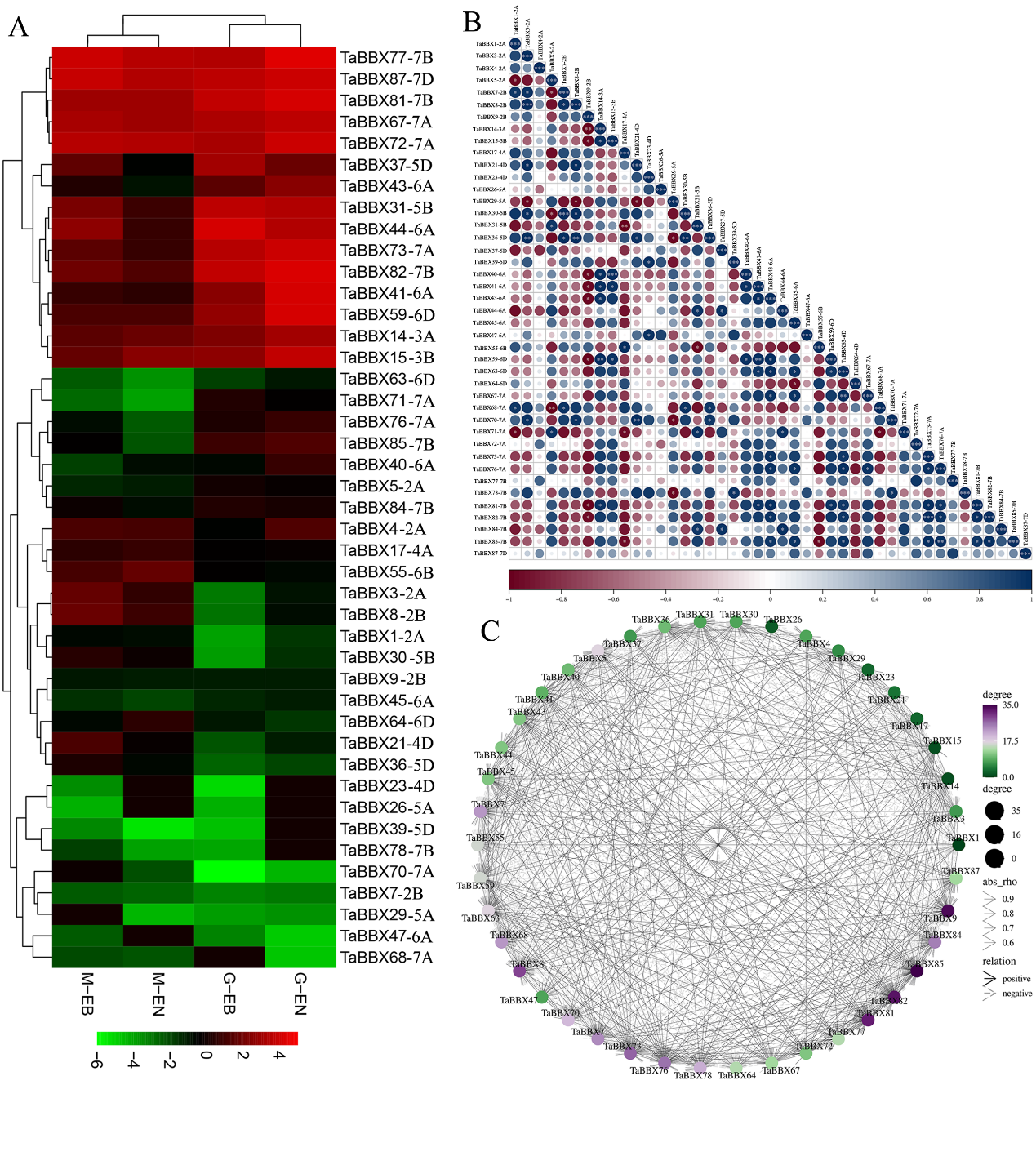


Fig.S9 Expression patterns of *TaBBXs* among GEO database.

A. Heatmap shows the hierarchical clustering of *TaBBXs* among GSE118474. Abbreviations represent Zhou 8425B embryo and endosperm collected time: G-EB, 14 DAP Embryo; M-EB, 25DAP Embryo; G-ES, 14 DAP Endosperm; M-ES, 25DAP Endosperm.

B. Correlation analysis using the R package program. Each correlation is shown by the shades of blue and red and the size of the circle shape. Blue and red indicate a positive correlation and negative correlation, respectively.

C. Co-regulatory networks. The co-regulatory networks of *TaBBXs* were established based on the PCCs of these gene pairs using transformed qPCR data. Different colors and styles indicate the different significance levels of the co-regulated gene pair


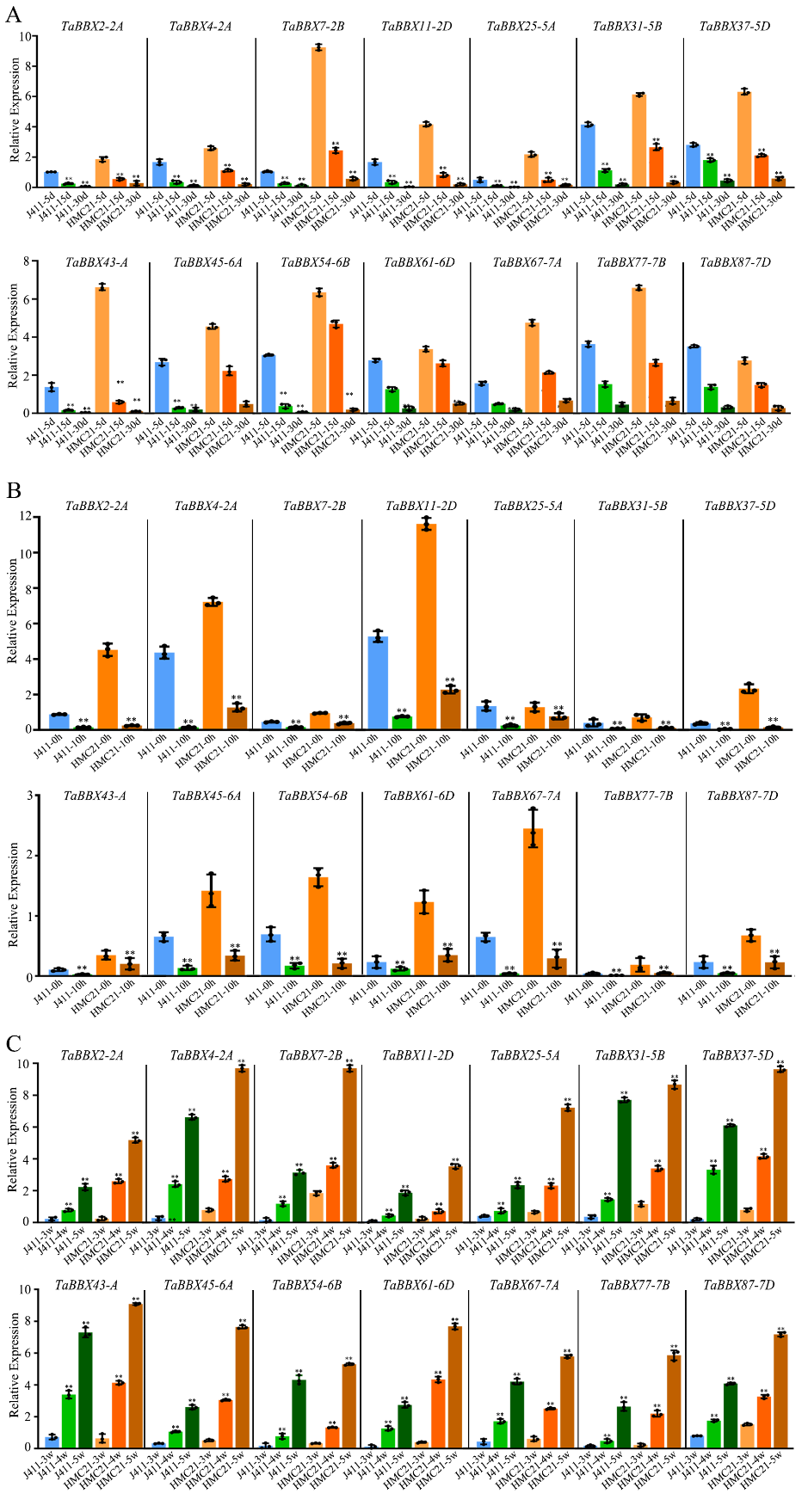


Fig.S10 Expression patterns of TaBBXs at different imbibition stages by qRT-PCR.

A. Expression patterns of TaBBXs at the 3w/4w/5w post anthesis. Three biological replicates per organization.

B. Expression patterns of TaBBXs after the 0 h and 10 h imbibition treatments, with 0 h as the control. Three biological replicates per organization.

C. Expression patterns of TaBBXs at 5, 15, and 30d post-harvest in the seeds of two wheat varieties Jing 411 (J411) and Hongmangchun 21 (HMC21). Three biological replicates per organization.

**Supplemental table**

Table S1 Primers for *TaBBXs*

Table S2 Detailed information about the predicted *TaBBXs*

Table S3 Amino acid composition of TaBBXs

Table S4 Detailed information about the *OsBBXs* and *AtBBXs*

Table S5 Ka, Ks and Ka/Ks ratios of Paralogous and orthologous pairs

Table S6 Groups of homoeologous *TaBBXs*

Table S7 Conserved domains composition of *TaBBXs*

Table S8 Subcellular localization prediction of TaBBXs

Table S9 Signal peptide analysis of *TaBBXs*

Table S10 miRNA analysis of *TaBBXs*

Table S11 Promoter analysis of the *TaBBXs*

Table S12 Gene ontology (GO) annotation analysis in *TaBBXs*

Table S13 Microarray data and Transcriptome data for *TaBBXs*
